# Sox10 contributes to the balance of fate choice in dorsal root ganglion progenitors

**DOI:** 10.1371/journal.pone.0172947

**Published:** 2017-03-02

**Authors:** Mariana Delfino-Machín, Romain Madelaine, Giorgia Busolin, Masataka Nikaido, Sarah Colanesi, Karen Camargo-Sosa, Edward W. P. Law, Stefano Toppo, Patrick Blader, Natascia Tiso, Robert N. Kelsh

**Affiliations:** 1 Department of Biology and Biochemistry and Centre for Regenerative Medicine, University of Bath, Bath, United Kingdom; 2 Centre de Biologie du Développement (CBD, UMR5547), Centre de Biologie Intégrative (CBI), Université de Toulouse, CNRS, UPS, Toulouse, France; 3 Department of Biology, University of Padova, Padova, Italy; 4 Department of Molecular Medicine, University of Padova, Padova, Italy; Institute of Molecular and Cell Biology, SINGAPORE

## Abstract

The development of functional peripheral ganglia requires a balance of specification of both neuronal and glial components. In the developing dorsal root ganglia (DRGs), these components form from partially-restricted bipotent neuroglial precursors derived from the neural crest. Work in mouse and chick has identified several factors, including Delta/Notch signaling, required for specification of a balance of these components. We have previously shown in zebrafish that the *Sry-*related HMG domain transcription factor, Sox10, plays an unexpected, but crucial, role in sensory neuron fate specification *in vivo*. In the same study we described a novel Sox10 mutant allele, *sox10*^*baz1*^, in which sensory neuron numbers are elevated above those of wild-types. Here we investigate the origin of this neurogenic phenotype. We demonstrate that the supernumerary neurons are sensory neurons, and that enteric and sympathetic neurons are almost absent just as in classical *sox10* null alleles; peripheral glial development is also severely abrogated in a manner similar to other *sox10* mutant alleles. Examination of proliferation and apoptosis in the developing DRG reveals very low levels of both processes in wild-type and *sox10*^*baz1*^, excluding changes in the balance of these as an explanation for the overproduction of sensory neurons. Using chemical inhibition of Delta-Notch-Notch signaling we demonstrate that in embryonic zebrafish, as in mouse and chick, lateral inhibition during the phase of trunk DRG development is required to achieve a balance between glial and neuronal numbers. Importantly, however, we show that this mechanism is insufficient to explain quantitative aspects of the *baz1* phenotype. The Sox10(baz1) protein shows a single amino acid substitution in the DNA binding HMG domain; structural analysis indicates that this change is likely to result in reduced flexibility in the HMG domain, consistent with sequence-specific modification of Sox10 binding to DNA. Unlike other Sox10 mutant proteins, Sox10(baz1) retains an ability to drive *neurogenin1* transcription. We show that overexpression of *neurogenin1* is sufficient to produce supernumerary DRG sensory neurons in a wild-type background, and can rescue the sensory neuron phenotype of *sox10* morphants in a manner closely resembling the *baz1* phenotype. We conclude that an imbalance of neuronal and glial fate specification results from the Sox10(baz1) protein’s unique ability to drive sensory neuron specification whilst failing to drive glial development. The *sox10*^*baz1*^ phenotype reveals for the first time that a Notch-dependent lateral inhibition mechanism is not sufficient to fully explain the balance of neurons and glia in the developing DRGs, and that a second Sox10-dependent mechanism is necessary. Sox10 is thus a key transcription factor in achieving the balance of sensory neuronal and glial fates.

## Introduction

The neural crest is a fascinating cell-type due to the diversity of cell-types derived from it, including diverse peripheral neurons, all peripheral glia, multiple pigment and skeletal cell-types, and various adult stem cells, including those for adult pigment cells[1]. One key question in development is how cells of different types are produced in the correct numbers, and the neural crest has been an excellent model system for addressing this issue [2]. Originating at the boundary of the neural plate and the non-neural ectoderm, neural crest cells delaminate from the dorsal neural tube, before undergoing extensive migration throughout the body [3, 4]. Among the derivatives of those migrating on the medial migration pathway between the spinal cord and the somites are the dorsal root ganglia (DRGs), consisting of both peripheral neurons and satellite glia, together with the Schwann cells that cover the peripheral nerve axons.

Work in both mouse and zebrafish has shown that the *Sry-related HMG box 10 (Sox10)* gene is crucial for the specification of all non-skeletogenic fates from the neural crest, which are absent or strongly reduced in numbers in strong loss-of-function mutants[5–13]. In each case, SOX10 drives expression of lineage-specific transcription factors that control the differentiation of the individual cell-types; in the *Sox10* mutants, transcription of these factors is severely reduced[14]. This process is best characterised in the melanocyte, where SOX10 drives expression of *microphthalmia-related transcription factor (Mitf; mitfa* in zebrafish), which encodes a basic Helix-Loop-Helix Leucine Zipper transcription factor and is a master regulator for melanocyte development[10, 11, 15–19]. For all glial cells, the lineage-specific transcription factors include SOX10 itself and Pax3[14, 20–23]. In the case of the DRG neurons, fate specification depends upon transcriptional activation of *neurogenin1 (neurog1)* and *neurog2*, which encode bHLH transcription factors[24–29], likely acting with other factors[30]. The role for Sox10 in activating *neurog1* has been somewhat controversial. In mouse, careful studies of the DRG phenotype of the loss-of-function mutants focussed on the glial phenotype; the DRG sensory neuron phenotype was interpreted as a secondary consequence of the failure of glial differentiation[20, 31]. In contrast, our studies in zebrafish showed a clear role for Sox10 in regulating early *neurog1* expression, and thus fate specification, which was strongly reduced in strong loss-of-function mutants [13].

The related question of how the *balance* of sensory neuron and satellite glial cell numbers is achieved has also received attention. With Sox10 required for both fates, it seemed that other factors must be key. Sensory neuron specification, at least in mouse (but not zebrafish[32]), depends upon Wnt signaling driving transcriptional activation of *Neurog1/2*[33, 34]. Likewise, glial fate specification is stimulated by Neuregulin signaling[35, 36]. Neuregulin signaling mediated by ErbB3b/ErbB2 is also required for formation of DRGs in zebrafish, perhaps due to defects in medial pathway migration of neural crest cells[37, 38]. Signaling for sensory neuron specification, but not for other functions of ErbB3b/ErbB2 signaling including glial development, are mediated through a complex formed around the scaffold protein Sorbs3[39]. Sonic hedgehog signaling also plays a role in determining sensory neuron number [40]. A key role is played by Delta-NotchDelta-Notch signaling, via lateral inhibition [41]. In mouse neural crest stem cells, stimulation of Delta-Notch signaling drives specification of glial fate[42]. Studies in chick have shown that nascent sensory neurons express Delta1 while non-neuronal cells in the ganglia express high levels of *Notch1* [43]. As a consequence, in cells adjacent to those expressing Delta1 *Neurog1* expression is inhibited, neuronal differentiation is delayed and glial fate specification proceeds [25, 43]. Consistent with this, transgenic suppression of Notch signaling in the neural crest of mouse also results in a neurogenic phenotype[44, 45]. In zebrafish, where the initial DRGs are very small, each consisting of only 2–5 neurons and around 6–10 support cells in 5 days post fertilisation (dpf) larvae, substantial growth of the DRGs occurs during larval development[46]. Recently, expression of *notch1a*, *deltaA* and *deltaD* has been noted in non-neuronal cells in DRGs[47]. Furthermore Delta-Notch signaling has been shown to contribute to the balanced production of neurons and glia during larval growth of the DRGs[47].

We identified the zebrafish *sox10*^*baz1*^ (hereafter referred to as *baz1*) mutant during a *sox10* allele screen [13]. Our initial observations showed that it combined features typical of other *sox10* mutant alleles, such as absence of normal melanocytes and of peripheral glial *myelin basic protein (mbp)* expression, and with a strong effect on xanthophores and iridophores that resembles, but is not quite as severe as in, a null allele (e.g. *sox10*^*m618*^, hereafter *m618*). Thus it appeared to be a strong hypomorph for the former cell-types, but a weaker hypomorph for the latter. More remarkably, certain neurons of the trunk and tail were more abundant than in wild-type siblings (a hypermorphic phenotype), in striking contrast to the dramatic reduction of these cells in all other *sox10* mutant alleles [13]. Sequencing identified the causal lesion as a single nucleotide substitution resulting in a V117M change in the HMG domain of the protein. Here, we provide a comprehensive characterisation of this unique *sox10* mutant phenotype. Our data demonstrate that the supernumerary neurons are DRG sensory neurons, that peripheral (as well as oligodendrocyte) glial fates are highly reduced, and that the neurons form precociously in a classic neurogenic phenotype. We show that this DRG sensory neurogenic phenotype can be partially reproduced by chemical manipulation of Delta-Notch signaling in early neural crest development. Consistent with this, we show expression of *delta* genes in nascent DRGs and *in vivo* activation of Notch signaling in non-neuronal cells in these early DRGs. Thus, Delta-Notch signaling plays a role in generating the balance of derivatives in the early DRGs. However, we show that changes in Notch signaling alone cannot explain the quantitative aspects of the *baz1* phenotype. *In silico* modelling suggests that the V117M mutation is likely to change the flexibility of the DNA-binding HMG domain, consistent with the fate-specific differences in the severity of the Sox10(baz1) phenotype. Importantly, we show that Sox10(baz1), like Sox10(Wild-Type, WT), but in contrast to classic loss-of-function Sox10(m618), can readily activate the *neurog1* promoter in zebrafish embryos. Consistent with this, ectopic expression of *neurog1* in a *sox10* loss-of-function context rescues the sensory neuronal phenotype. We propose that the *baz1* DRG phenotype results from severe disruption of the process of glial specification, whilst remarkably sparing sensory neuron specification; we suggest that a crucial aspect of Sox10-dependent glial specification is a Notch-*independent* repression of *neurog1* expression that acts alongside Delta-Notch signaling in these nascent sensory neurons to prevent supernumerary neuronal differentiation. The *baz1* mutant DRG phenotype indicates that Sox10 is important not just for specification of DRG cell-fates, but also for the balance in the production of both neurons and glia.

## Results

### Supernumerary neurons in Sox10baz1 mutants express sensory, but not sympathetic or enteric markers, and form precociously

**Fig 1:**
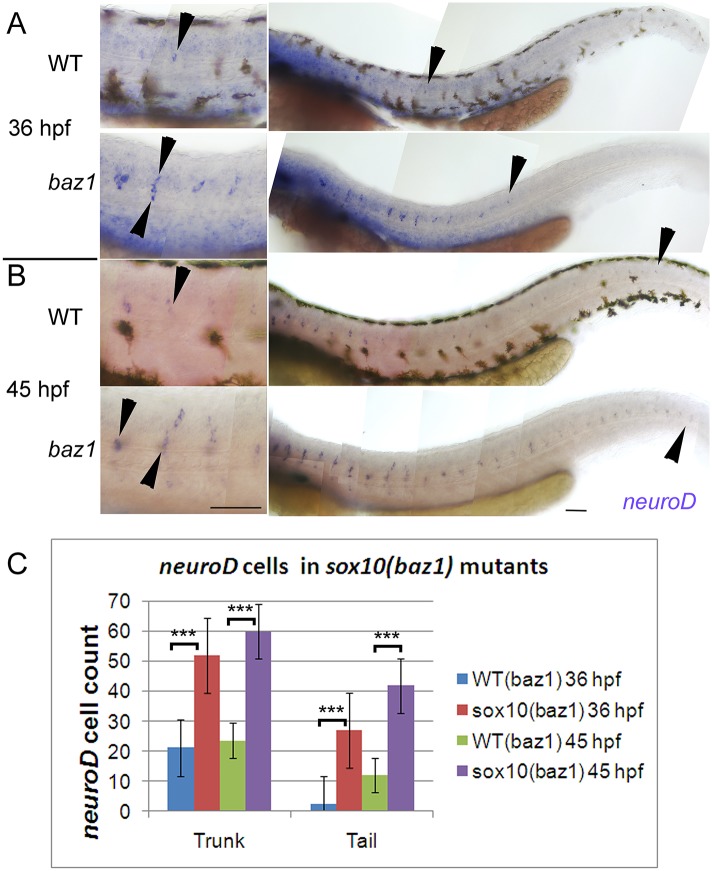
Precocious and supernumerary sensory neuron specification in *sox10*^*baz1*^ mutants. A,B) *neurod1* expression is seen in more cells (close-ups in left panels; arrowheads indicate a subset of *neurod1*^*+*^ cells) and extending more posteriorly (right panels; arrowhead marks posteriormost *neurod1*^*+*^ DRG) in *baz1* mutants compared with WT siblings at both 36 and 45 hpf. C) Counts of *neurod1*^*+*^ cells on one side of embryo at 36 and 45 hpf embryos (N = 11 for all conditions except 36 hpf *baz1*, where N = 13). *baz1* mutants significantly different to WT siblings (Student’s *t* test; ***, p<0.0001. In this and all subsequent images, embryos are shown in lateral view with dorsal to the top and anterior to the left, unless otherwise stated. Scale bar, 100 μm.

In our initial description of the *sox10*^*baz1*^ phenotype (hereafter referred to as *baz1*), we showed that at 5 days post-fertilisation (dpf) embryos homozygous for this allele display an approximately 2-fold increase in Elav1/Hu-expressing neurons relative to WT siblings at locations where dorsal root ganglia (DRG) develop, and that this phenotype could be detected as early as 36 hours post-fertilisation (hpf) by monitoring the expression of the proneural gene *neurog1*[13]. To address the mechanisms underlying this surprising phenotype, we first sought to confirm that the supernumerary neurons are indeed DRG sensory neurons. Like *neurog1*, expression of *neuronal differentiation1* (*neurod1*) labels the sensory neuron lineage [24, 48]. In *baz1* mutant embryos, *neurod1* expression was seen in a substantially increased number of cells on the medial pathway, in a pattern that strikingly resembled that of Elav1/Hu (Fig 1; [13]). Counts demonstrated that *neurod1+* cells were increased around 3-fold compared with WT siblings (Fig 1C). Furthermore, *neurod1* expression was seen precociously, with expression in mutants extending further posteriorly (i.e. to developmentally younger regions of the embryo) at both 36 and 45 hpf (Fig 1A and 1B, right panels). Similarly, supernumerary cells in *baz1* mutants were clearly *islet1*-positive (S1 Fig), consistent with them being specified as sensory neurons.

**Fig 2:**
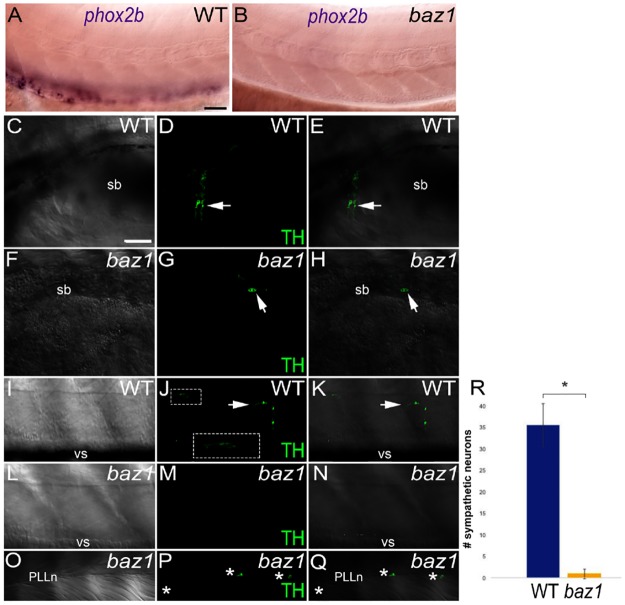
Enteric precursors are absent in baz1 mutants and supernumerary neurons in trunk and tail do not express markers of non-sensory neuronal types. A,B) *phox2b* expression at 72 hpf in wild-types (A) is restricted to the enteric neuron progenitors, but is absent from *baz1* embryos (B). Tyrosine hydroxylase (TH) immunofluorescence reveals differentiated sympathetic neurons at 7 dpf in WT (C-E, I-K), whereas these are much reduced in *baz1* mutants (F-H, L-M). White arrows indicate a subset of TH^+^ neurons. Inset in J represents enlargement of boxed area to show autonomic neuron chain more clearly. Occasional *baz1* mutants show anti-TH immunofluorescence in neurons (*) associated with the PLLn (O-Q). DIC (C,F,I,L,O) and immunofluorescent TH images (D,G,J,M,P) are merged in panels E,H,K,N,Q respectively. sb, swim bladder; vs, ventral stripe. R) Quantitation (mean ± s.d.) of anti-TH positive sympathetic neurons in WT (N = 16) and *baz1* mutants (N = 16). * indicates significant difference (two-tailed *t* test, p<0.001) Scale bar, 50 μm(C).

In contrast to sensory neuron markers, markers of other NCC-derived peripheral neurons were absent both at their endogenous site of expression and along the medial pathway (Fig 2). We had previously shown that Elav1/Hu+-enteric neurons were lacking in *baz1* mutant embryos [13]. Here, we complement this observation by showing that *phox2b*-expressing enteric progenitors were also missing from the developing gut of *baz1* mutants at 72 hpf, and no sign of ectopic *phox2b* expression was detected on the medial pathway (Fig 2A and 2B). Likewise, immunofluorescent detection of the sympathetic neuron marker Tyrosine Hydroxylase (TH) was strongly reduced in *baz1* mutants at 7 dpf with no expression on the medial pathway (Fig 2C–2N, quantified in R); unexpectedly, embryos occasionally showed ectopic TH-expressing neurons associated with the Posterior Lateral Line nerve (PLLn; Fig 2L–2N). These data strongly suggest that the supernumerary neurons detected on the medial pathway of *baz1* mutant embryos are restricted to the sensory lineage.

**Fig 3:**
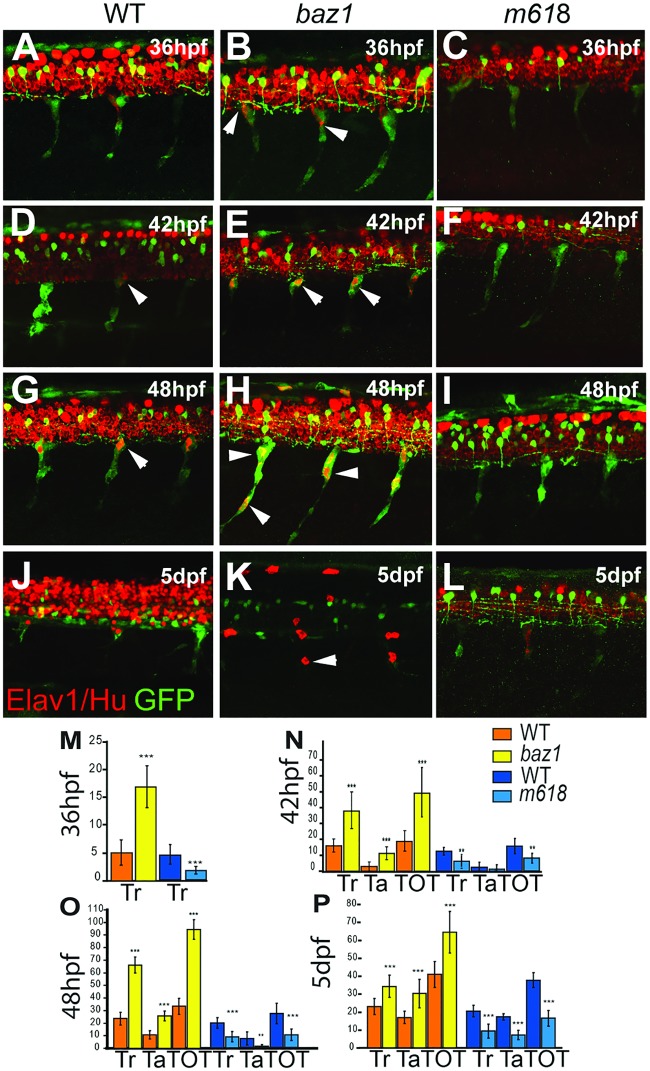
Medial pathway neural precursors undergo precocious and supernumerary differentiation into neurons in baz1 mutants. Confocal images of developing trunk DRGs of WT (A, D, G, J), *baz1* (B, E, H, K) and *m618* mutants (C, F, I, L) showing Elav1/Hu (red) and *sox10*:*GFP* (green) at each of 36 (A-C), 42 (D-F), 48 hpf (G-I) and 5 dpf (J-L). Arrowheads indicate subset of Elav1/Hu^+^ DRG sensory neurons. M-P) Counts (mean±s.d.) of trunk (Tr) and tail (Ta) and total (TOT) Elav1^+^ cells in DRGs of *baz1* (yellow) and *m618* (blue) mutants and their respective WT siblings. Significantly elevated numbers of neurons are indicated (two-tailed Student’s *t* test; **, p<0.01; ***, p<0.001). Note in panels J-L) that variable prominence of Elav1/Hu detection in spinal cord is an artefact of antibody penetration into CNS at this late stage. Scale bar, 50 μm.

To explore further the temporal aspects of the formation of these supernumerary sensory neurons we compared the distribution of Elav1/Hu antigen with *Tg(sox10(4*.*9)*:*GFP)*^*ba2*^, in which GFP initially labels all neural crest cells, including neural progenitors on the medial pathway, but later is maintained only in glial cells (Fig 3; [13]). Using GFP as a short-term lineage marker for neural crest-derived DRG progenitors, we noticed that in WT embryos Elav1/Hu immunofluorescence was seen in only a few GFP+ cells in the anterior trunk by 36 hpf, and not until 48 hpf were substantial numbers detected posteriorly (Fig 3A, 3D and 3G; quantified in panels M-O). At these stages many Elav1/Hu-;GFP+ progenitors were seen on the medial pathway. In *sox10*^*m618*^ mutants (hereafter referred to as *m618*), reduced numbers of cells express Elav1/Hu, and most cells remain Elav1/Hu-;GFP+ (Fig 3C, 3F and 3I). In contrast, *baz1* mutants show substantial numbers of Elav1/Hu+;GFP+ double positive cells in both the anterior and posterior trunk from 36 hpf onwards, and by 48 hpf have approximately three times as many as stage-matched WT siblings, although we note that not all GFP+ cells express Elav1/Hu (Fig 3B, 3E, 3H and 3M–3O). By 5 dpf, the GFP signal is often strongly reduced, especially in *sox10* mutants, due to a failure of the GFP to persist in unspecified precursors; nevertheless, DRG neurons are still distinctive due to their combination of spatial localisation and Elav1/Hu immunofluorescence. Elevated numbers of neurons remain, but there are now fewer than at earlier stages (Fig 3J–3L and 3P).

To confirm these observations, we performed time-lapse studies of neuronal specification in *baz1* mutant embryos and their WT siblings. We generated fish carrying the *Tg(-4*.*9sox10*:*GFP)*^*ba2*^ and *Tg(-8*.*4neurog1*:*nRFP)*^*sb3*^ transgenes and bred them onto the *baz1* mutant background [13, 49]. In these fish, early neural crest cells (NCCs), including the nascent DRG, are labelled with GFP and neuronal specification is indicated by the initiation of RFP expression in a subset of these cells (S1 and S2 videos; S2 Fig); time-lapse focused on the posterior trunk, and typically began at around 36 hpf and continued for up to 25 hours. In WT fish, RFP expression was not normally detected in NCCs at the beginning of the analysis, but isolated sensory neurons were specified during the next day of development. In contrast, *baz1* mutants regularly showed RFP expression in sensory neurons from the beginning of the time-lapse and multiple NCCs in each ganglion begin expressing RFP over the course of the analysis.

Finally, we addressed whether a change in the balance of proliferation or apoptosis in the DRG could account for the increased numbers of sensory neurons in *baz1* mutant embryos. Apoptosis rates were assessed by TUNEL in which DRG cells were identified by GFP expression from the *Tg(-4*.*9sox10*:*GFP)*^*ba2*^ transgene at 36 and 48 hpf in *baz1* mutants and their WT siblings. Although TUNEL+ cells were detected in embryos at all stages, apoptosis rates in the DRGs were negligible in either genetic context (Table 1). Likewise, proliferation rates in DRG were determined using phospho-Histone H3 (pH3) immunofluorescence in *baz1* mutants and their WT siblings at 36, 42 and 48 hpf; DRG cells were identified by GFP fluorescence from the *Tg(-4*.*9sox10*:*GFP)*^*ba2*^ transgene (36 hpf) or anti-Elav1/Hu immunofluorescence (42 and 48 hpf). In all cases, although pH3+ cells were detected widely in these embryos, proliferation rates in the DRGs were also negligible (Table 2).

**Table 1:** Apoptosis in the DRGs is negligible in *baz1* mutants and their WT siblings.

	WT			*baz1*		
Stage (hpf)	No. Embryos	Total No. DRGs assessed	Total no. GFP+;TUNEL+ DRG cells	No. Embryos	Total No. DRGs assessed	Total no. GFP+;TUNEL+ DRG cells
36	15	195	1	10	130	3
48	10	250	0	8	200	0

Table shows number of embryos scored, number of DRGs assessed per embryo and total number of TUNEL+ DRG cells in all embryos scored.

**Table 2:** Proliferation in the DRGs is negligible in *baz1* mutants and their WT siblings.

	WT				*baz1*			
Stage (hpf)	No. Embryos	Total No. DRGs assessed	Total no. GFP+;pH3+ DRG cells	Total no. Elav1/Hu+;pH3+ DRG cells	No. Embryos	Total No. DRGs assessed	Total no. GFP+;pH3+ DRG cells	Total no. Elav1/Hu+;pH3+ DRG cells
36	22	286	6	nd	8	104	1	nd
42	16	320	nd	2	3	60	nd	0
48	15	375	nd	3	5	125	nd	2

Table shows number of embryos scored, number of DRGs assessed per embryo and total number of pH3+ DRG cells in all embryos scored. nd, not determined

Taken together, these data suggest that the supernumerary neurons detected in the DRG of *baz1* mutant embryos at 48 hpf are specified sensory neurons, whereas in WT siblings at the same stage many NCCs appear to remain in a progenitor state. We also conclude that the balance of proliferation and apoptosis in the DRG is unlikely to contribute significantly to the phenotype observed in *baz1* mutants.

In contrast to the unique sensory neuron phenotype, *baz1* mutants show a severe defect in glial fate specification and differentiation that is comparable to, but slightly weaker than, that of other *sox10* alleles we have studied ([50]; S3 Fig). Thus, *sox10* expression associated with the trunk medial pathway in *baz1* mutants showed a similar pattern to WT, and was more prominent than in *m618* mutants (compare S3 Fig panel C with panels G and K, white asterisks); interestingly, the developmentally younger DRGs in the tail more closely resembled those in *m618* (compare S3 Fig panels D with H and L, white asterisks). Not withstanding the mutant nature of the proteins encoded by these transcripts, the fact that *sox10* transcripts are detectable enables us to use them as a marker of *sox10*-expressing cell states, including neural progenitor cells (early) and differentiating glia (later). We interpret these data to suggest that *sox10* expression is initially normal, but then fails to be maintained in putative glia in the *sox10* mutant alleles, and that this loss is more pronounced in *m618*. We also saw a very strong reduction in the number of *foxd3*-expressing Schwann cell precursors at 48 hpf on both the spinal nerves (S3 Fig panels M,P) and the PLLn (S3 Fig panels N,Q), with remaining cells on the PLLn concentrated near the base of the nerve (S3 Fig panel Q). Absence of peripheral glia on the PLLn results in supernumerary production of lateral line neuromasts [51]; using *isl1* as a marker for neuromasts [52], we saw that *baz1* mutants also showed precocious differentiation of neuromasts (S3 Fig panels O,R), confirming the absence of PLLn Schwann cells. In summary, our analysis shows a consistent picture of reduced glial cell development, with much reduced numbers of peripheral glial cells and no sign of Schwann cell differentiation.

In addition, oligodendrocyte differentiation is impaired in *baz1* mutants. *Sox10* expression also marks oligodendrocyte precursors and differentiating oligodendrocytes, and Sox10 is required for oligodendrocyte differentiation. We asked whether *baz1* mutants showed defects in oligodendrocyte development. Using *sox10* expression in the ventral CNS as a marker of oligodendrocyte precursors, and dispersed expression in scattered cells throughout the CNS as a marker of oligodendrocytes, we saw no change in oligodendrocyte precursors and specification in *baz1* mutants (S4 Fig panels A-F). In contrast, *mbp* expression in differentiating oligodendrocytes is reduced, indicating that oligodendrocyte differentiation is impaired in *baz1* mutants (S4 Fig panels G,H).

### Supernumerary neurons and Notch signaling

The sensory neuron phenotype we describe in *baz1* mutants shows the hallmarks of a neurogenic phenotype, with supernumerary and precocious neuronal differentiation at the expense of glial cell-types (Fig 3, S2 Fig; [13]). Classically, such phenotypes result from disruption of Notch signaling. To explore a potential role for Notch signaling in DRG development, we first asked if the expression of Notch pathway components could be detected in the nascent DRGs. For this, we explored the patterns of expression of *jagged* and *delta* ligands at stages from 24 to 38 hpf, focusing on the posterior trunk region; overlapping expression with neural crest cells in the nascent DRG was identified by their co-expression of *sox10*, *foxd3* or *neurog1*. As expected, the expression of *jagged* genes was generally detected at low levels. While we confirmed the previously published patterns of expression, for instance of *jag1b* and *jag2* in pronephric duct, notochord and spinal cord, we did not see pronounced expression of *jag1a*, *jag1b* or *jag2* in nascent DRGs (data not shown), consistent with the findings of Zecchin et al [53]; over-developing the *in situ* hybridisations highlighted a low level expression of *jag1a* expression in cells adjacent to, but not overlapping with, the nascent DRGs (data not shown).

**Fig 4:**
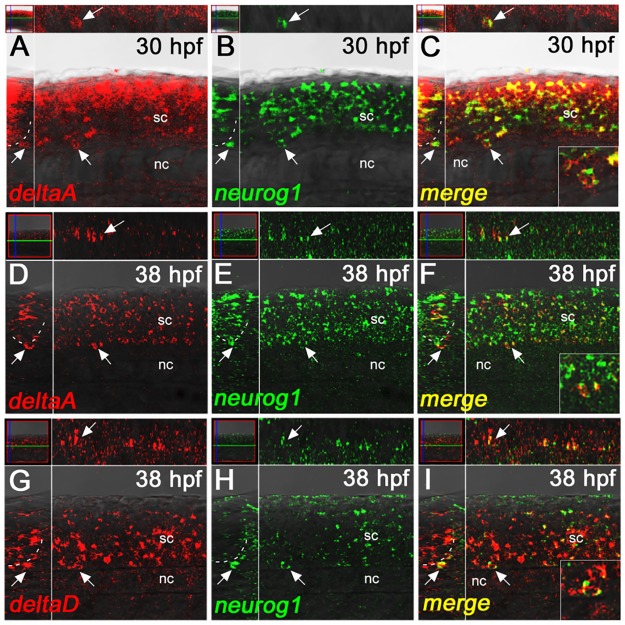
deltaA and deltaD gene expression overlaps with neurog1 in the nascent DRGs. A-C) *deltaA* expression (red) clearly overlaps with *neurog1* (green) in the nascent DRG (arrows) at 30 hpf. D-F) At 38 hpf, *deltaA* expression is clearly seen in the DRGs, but weaker signals make it difficult to discern if expression is in the same cells as express *neurog1* or simply in other cells of the ganglia. G-I) *deltaD* expression (red) clearly overlaps with *neurog1* (green) in the nascent DRG (arrows) at 38 hpf. All main panels are confocal images of fluorescent dual-color *in situ* hybridisations in lateral view, with insets showing *y-z* planes (left) and *x-z* planes (above) for each. Insets in the bottom right of panels C, F and I show enlargements of the double-labeled cells indicated by the arrows. nc, notochord; sc, spinal cord.

In contrast, we found clear expression of *delta* ligands in the nascent DRGs, identified by *neurog1* expression. We examined all four *delta* genes, but found that only two (*deltaA* and *deltaD*), showed overlapping or adjacent expression with *neurog1*-positive DRG cells, consistent with recently published data [47]. At 30 hpf, *deltaA* and *neurog1* showed strong colocalisation in individual cells of the nascent DRG (Fig 4A–4C and data not shown). At later stages (shown at 38 hpf in Fig 4D–4F), *deltaA* and *neurog1* remain expressed in the nascent DRGs, but only weakly, preventing unequivocal assessment of co-localisation at the single cell level. Similarly, *deltaD* expression showed co-localisation with *neurog1* in individual cells of the nascent DRGs at these same stages (Fig 4G–4I and data not shown). We confirmed these observations at 30 hpf for the *deltaA* and *deltaD* genes using colocalisation with the alternative DRG marker, *neurod1* (S5 Fig). We conclude that Delta-Notch signaling, mediated through one or both of DeltaA or DeltaD, might contribute to fate specification within zebrafish DRG.

**Fig 5:**
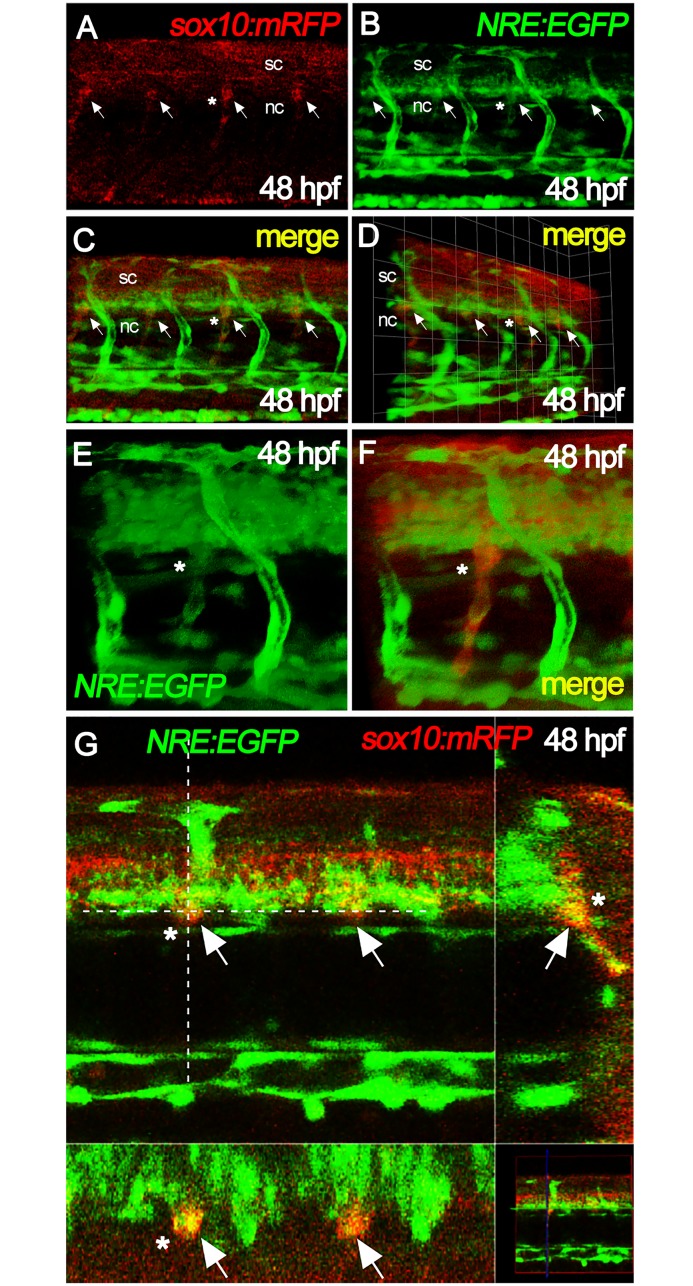
Notch signaling is active in nascent DRGs. Neural crest cells in the DRGs of 48 hpf embryos (arrows) were readily identified by expression of mRFP reporter in *sox10*:*mRFP* fish (A). These overlapped with eGFP expression from the *12xNRE*:*eGFP* (Notch signaling) reporter (B), as shown in superimposed image (C and D). Close-ups of individual DRGs labelled with asterisk are shown in panels E and F. Panel G shows DRGs (white arrows, yellow signals) as single plane acquisitions, in lateral view (top left), transversal view (top right), dorsal view (bottom left) and mini global view (bottom right). Note that in all panels eGFP is also strongly visible in blood vessels, as expected for the Notch reporter. nc, notochord; sc, spinal cord.

We then asked whether DRG cells were responding to these ligands by activating the Notch pathway. For this, we used the Notch reporter line, *Tg(12xNRE*:*eGFP)*, bearing twelve Notch Responsive Elements (NRE) upstream of the coding sequence of eGFP [54]. DRG cells were identified using a *Tg(sox10(7*.*2)*:*mRFP)* reporter line [55]. As previously described for another Notch reporter line, strong eGFP expression is detected in all blood vessels [56], but we also see weaker signals overlapping the DRG (arrows and asterisk in Fig 5A–5G). While co-expression of eGFP and RFP was best seen at 48 hpf, overlap between the Notch-reporter and the DRG marker was detected as early as 36 hpf (Fig 5A–5G; data not shown). We conclude that Delta-Notch signaling is active within neural crest cells of the nascent DRG in zebrafish embryos starting at a stage before 36 hpf. These data are fully consistent with the *delta* expression patterns reported above and with the idea that DRG neuronal numbers are regulated, at least in part, by this pathway.

**Fig 6:**
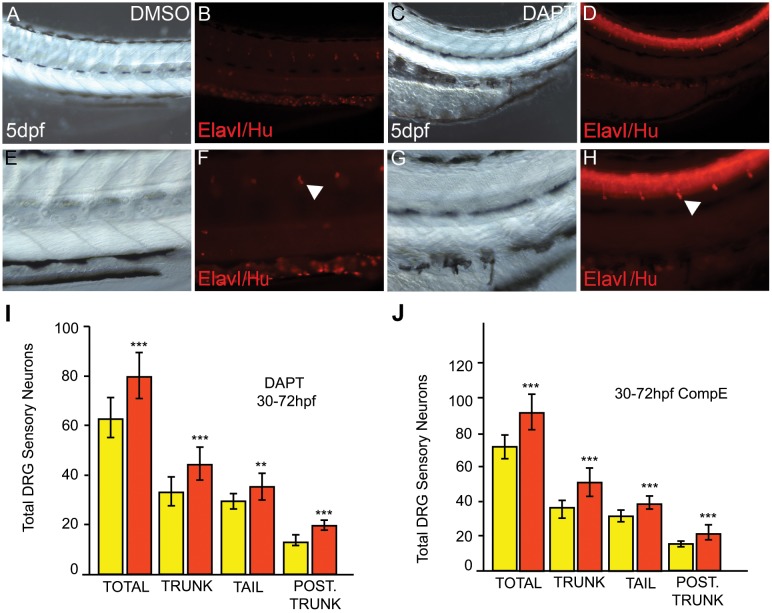
Notch inhibition phenocopies the sox10baz1 DRG phenotype. A-H) Posterior trunks of WT embryos treated with 100 μM DAPT from 30–72 hpf (C,D,G,H) and controls exposed to DMSO carrier alone (A,B,E,F). E-F show enlargements of A-D. Treated embryos show dorsally-curved body axis (C). Immunofluorescence for Elav1/Hu revealed increased numbers of DRG sensory neurons (arrowhead). I,J) Quantitation of DRG sensory neurons in 5 dpf embryos after treatment with 100 μM DAPT (I, orange) or 100 μM Compound E (J, orange) from 30–72 hpf; compared with control DMSO-treated embryos (yellow in I and J), sensory neuron numbers are significantly increased (***) when Notch signaling is inhibited. While trunk refers to the entire somitic region from posterior to the otic vesicle to the anus, tail refers to the post-anal body; posterior trunk refers to the 7 most posterior somatic segments dorsal to the yolk sac extension. N = 13 (Control, I), 13 (DAPT, I), 16 (Control DMSO, J) and 15 (Comp. E, J). Significance of elevated numbers of neurons was evaluated by two-tailed Student’s *t* test (**, p<0.01; ***, p<0.001).

Finally, we asked whether loss of Notch signaling phenocopies the *baz1* DRG phenotype. Classical zebrafish mutants affecting Delta-Notch signaling show early defects in neural crest specification [27], precluding examination of later roles in DRG formation. Notch signaling depends upon γ-secretase-mediated cleavage of Notch receptor that can be blocked with DAPT, a γ -secretase inhibitor that has been widely used to study Notch function in zebrafish [57]. We, thus, asked whether DAPT treatment could modulate the number of DRG sensory neurons. We found that a 30–72 hpf treatment window (i.e. extending throughout the initial phase of sensory neuron specification) resulted in a significant increase in DRG sensory neurons in both trunk and tail, although the degree of increase was not as striking as in the *baz1* mutants (Fig 6A–6H, quantified in 6I; compare Figs 1 and 3). As reported before, treated embryos showed a bending of the antero-posterior axis (Fig 6C, compare Fig 6A), but are otherwise morphologically grossly normal. To verify the specificity of this effect, we used a second γ -secretase inhibitor, Compound E [58], which gave a quantitatively comparable increase in DRG sensory neuron numbers (Fig 6J). We conclude that DRG neuron number in zebrafish, as in mouse and chick, is regulated by Notch signaling, a conclusion also reached in a recent, independent study [47].

**Fig 7:**
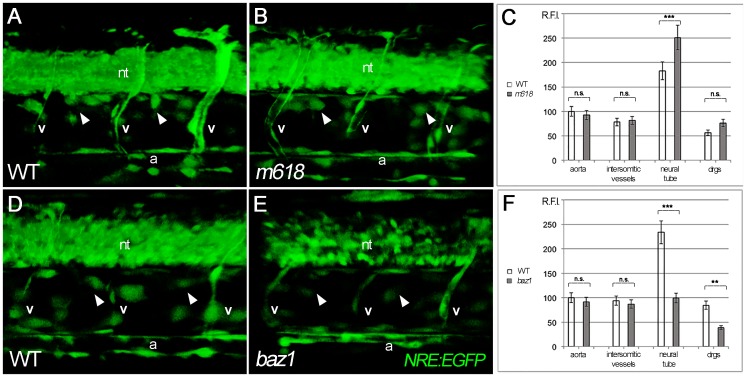
Quantification of Notch reporter differences in m618 and baz1 mutants at 48 hpf. A-F) confocal acquisitions of WT control (A,D) and mutant (B, *m618*; E, *baz1*) trunk regions, followed by Notch reporter (NRE:EGFP) fluorescence analysis (C,F). A slight increase of reporter signal is detected in the neural tube (nt) and dorsal root ganglia (drg, arrowhead) of *m618* mutants (B) compared to controls (A), while a decrease of signal is detected in the same regions of *baz1* mutants (compare E with D). Relative fluorescence intensity (RFI) in aorta (a) and intersomitic vessels (v) appears unmodified in all conditions. n = 6 measurements per condition. n.s. = not significant; ** = p<0.01; *** = p<0.001.

Implicit in the above analysis is the assumption that Notch signaling is affected in, and thus likely contributes to, the *baz1* mutant phenotype, whereas in *m618* mutants, Notch signaling was likely to remain intact. However, it is conceivable that Notch signaling might be unaffected in both mutant contexts, and that differences in another mechanism are solely responsible for the distinct sensory neuron phenotypes of *m618* and *baz1* mutants. To test our assumption, we evaluated Notch signaling in the DRGs of *m618* and *baz1* mutants, by crossing the *Tg(12xNRE*:*eGFP)* reporter onto the respective *sox10* mutant backgrounds. Assessing DRG cells at 48 hpf, while we consistently observed Notch reporter activity in *m618* mutants (Fig 7B), DRG signals were considerably weakened in *baz1* mutants (Fig 7E). Quantification of the fluorescence intensity from the DRGs confirmed that Notch signaling in *baz1* mutants was reduced by around 50% (Fig 7F), whereas in *m618* mutants levels were not significantly changed compared with WT siblings (Fig 7C). Reduction of Notch signaling in baz1 and normal activation in m618 mutants was also observed using an additional Notch responsive line expressing a nuclear-localized red protein *Tg(EPV*.*Tp1-Mmu*.*Hbb*:*NLS-mCherry)*[59], thus confirming our previous results with an independently generated Notch reporter allele (data not shown). In all cases fluorescence from the dorsal aorta and intersomitic vessels was comparable between mutants and their respective WT siblings. We saw consistent reduction in Notch reporter expression in the neural tube of *baz1* mutants compared with WT siblings, whereas in *m618* mutants Notch signaling appeared, if anything, to be elevated compared to siblings (Fig 7B, 7C, 7E and 7F; S6 Fig). It is likely that the elevated levels in *m618* mutants may at least in part be an artefact of the lack of melanin (which will partially quench the fluorescent signal). However, the significantly lowered expression in *baz1* mutants is unexpected, but conceivably results from differences in oligodendrocyte progenitor development in these two different mutant alleles; this will require further investigation. Meanwhile, in the context of DRG development, we conclude that, as predicted, Notch signaling remains active in the DRGs of *m618* mutants, but is significantly reduced in *baz1* mutants. Analysis of *gfp* transcriptional expression in these lines, using *sox10* to label medial pathway neural crest cells in the nascent DRGs confirms this interpretation (S7 Fig).

### Sox10(baz1) protein retains transcriptional activity at the neurog1 locus

The *baz1* DRG phenotype is consistent with a decrease of Notch signaling. However, the quantitative difference in the increase in neuronal number in *baz1* compared with the maximum effect of chemical inhibition of Notch signaling suggested this is, at best, only a partial explanation. The *baz1* mutation results in a single amino acid substitution (V117M) in the DNA binding HMG domain [13]. Molecular dynamics simulation studies confirmed the expectation that the Sox10(baz1) substitution results in relatively subtle changes in the protein, most strikingly an apparent reduction in the flexibility of the HMG domain (S8 Fig). Whilst these observations will require detailed experimental confirmation, they are consistent with the hypothesis that Sox10(baz1) may show differential affinity for the regulatory sequences of target genes, retaining transcriptional activity at some target genes whilst losing activity at others.

**Fig 8:**
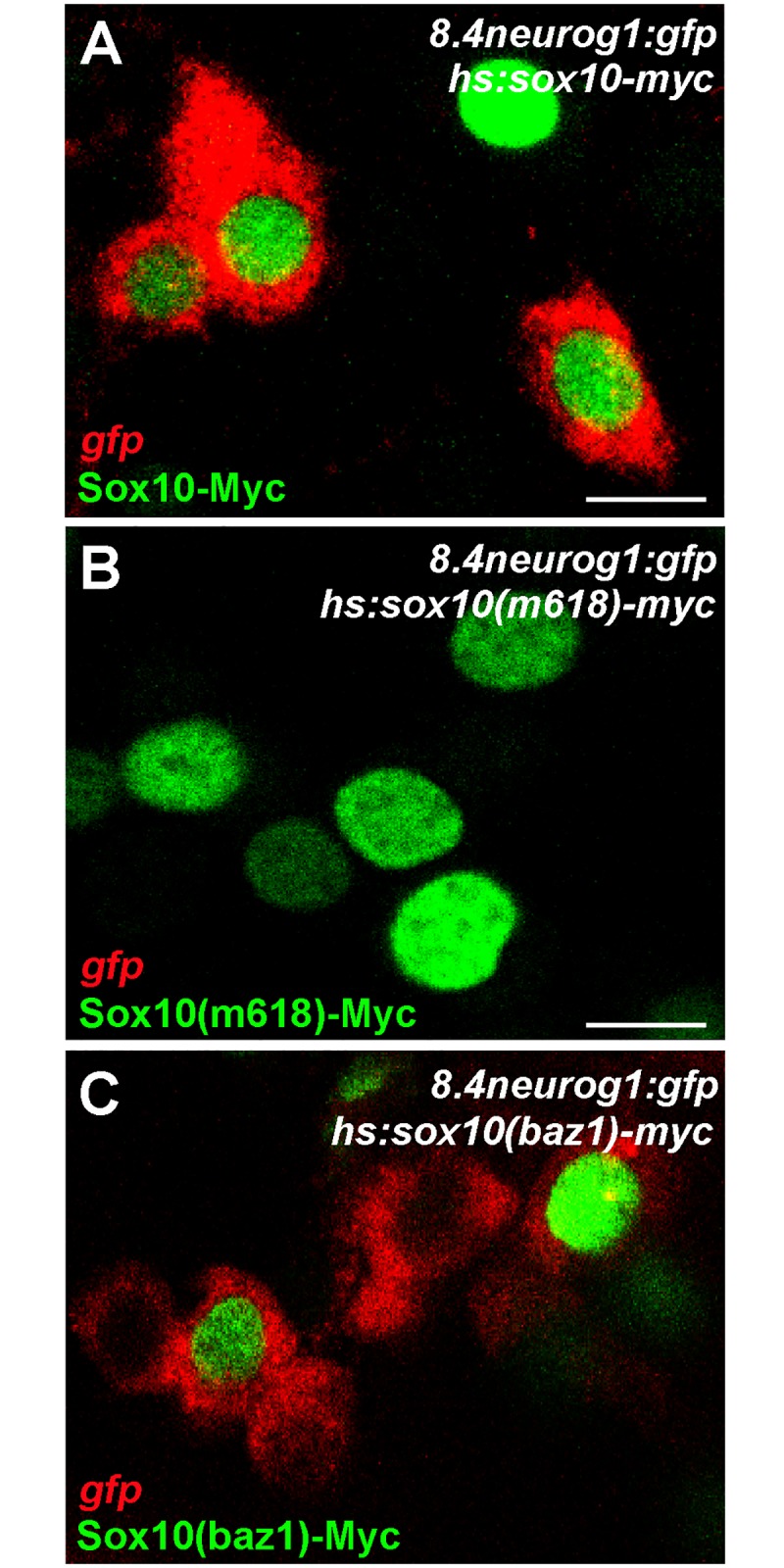
Sox10(baz1) retains transcriptional activity at neurog1. *Tg(8*.*4neurog1*:*GFP)* embryos were injected with expression plasmids encoding variants of Sox10. Embryos were heat-shocked at 7 hpf and fixed at 10 hpf. Expression of the Sox10 variant proteins was detected by immunofluorescent detection of their Myc tags (green) and activation of the *neurog1* reporter gene is seen by *In situ* hybridisation against the *gfp* mRNA (red). Note how both WT and baz1 proteins activate *Tg(8*.*4neurog1*:*GFP)* reporter expression (A C), whereas m618 does not (B). Apparent non-autonomous activation of *gfp* after expression of Sox10(baz1) but not Sox10(WT) nor Sox10(m618) was reproducibly seen, but always restricted to cells adjacent to those expressing Sox10(baz1); the mechanism underlying this remains unclear but could be related to differential stability of the various Sox10 proteins.

We have previously shown that null alleles of *sox10* display a substantial reduction in DRG sensory neurons that correlates with reduced expression of *neurog1* [13]; Neurog1 is the obligate proneural gene for DRG sensory neuron specification in zebrafish [29]. We have also shown that Sox10(WT) but not Sox10(m618) drives ectopic *neurog1* transcription when mis-expressed [13]. Given the generally strong loss-of-function nature of the *baz1* phenotype, the increase in *neurog1* expressing cells on the medial pathway in *baz1* embryos is particularly surprising. Since it seemed unlikely that this increase simply reflected a reduction in Notch activity, we asked whether Sox10(baz1) retains the ability to drive *neurog1* expression. To test this, we took advantage of a previously reported *Tg(-8*.*4neurog1*:*GFP)*^*sb1*^ transgenic line that largely replicates the endogenous neurog1 expression pattern in neuronal precursors, including the DRG sensory neurons [49]. Consistent with our published results, GFP transcription from the transgene was not driven by mis-expression of Sox10(m618) (Fig 8B). In contrast, Sox10(baz1) induced GFP transcription in a manner similar to Sox10(WT) (Fig 8A and 8C), indicating that the baz1 mutation does not affect Sox10 transcriptional activity at the *neurog1* locus. These results suggest that the increase in DRG sensory neurons seen in *baz1* mutants is not solely due to a reduction in Notch signaling.

### Neurog1 rescues sensory neurons in sox10 morphants

**Fig 9:**
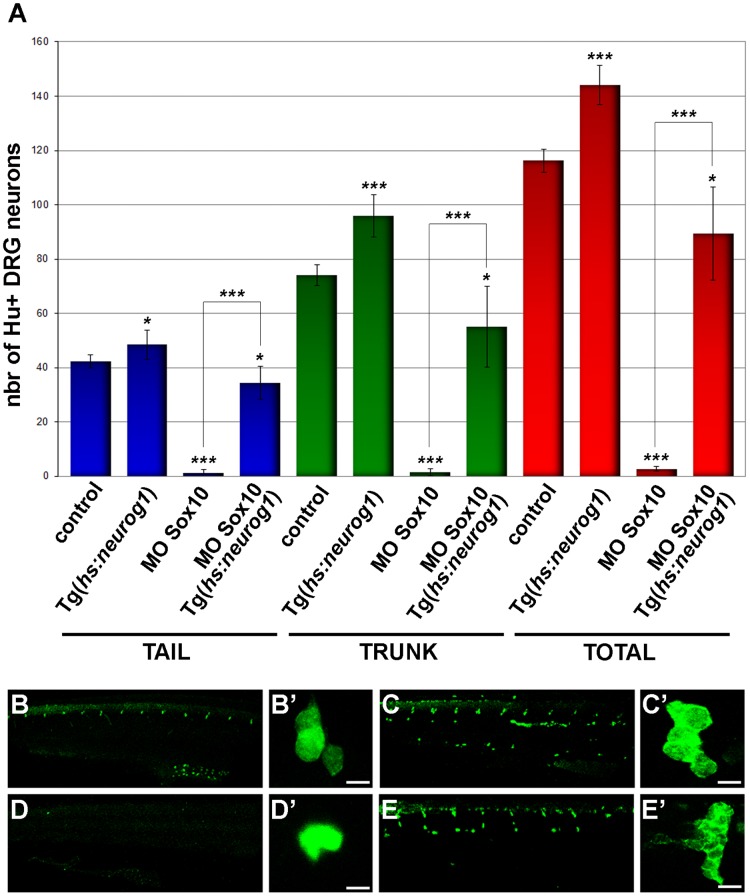
Global mis-expression of Neurog1 is sufficient to phenocopy the baz1 mutant DRG phenotype. (A) Quantification of mean (±s.d.) total numbers of Elav1/Hu+ DRG neurons in tail (blue), trunk (green) or total (red). The number of embryos analysed was 6 (WT control), 6 (*hs*:*ngn1*), 8 (MO *sox10*) and 7 (MO *sox10*, *hs*:*ngn1*). Significance of differences as determined by two-tailed Student’s *t* test comparisons is indicated by * (p<0.05), ** (p<0.001) and *** (p<0.0005). Control (B-C’) or *sox10*-morpholino injected (D-E’) embryos were left uninduced (B,B’,D,D’) or subjected to heat-shock (C,C’,E,E’) and subsequently processed for Elav1/Hu immunofluorescence at 5 dpf; embryos were heat-shocked at 28hpf for 1 hour. Panels show lateral views of trunk (B,C,D,E) and close-ups of representative DRGs (B’,C’,D’,E’).

Our data clearly indicated both that *neurog1* is precociously and ectopically expressed in nascent *baz1* DRG and that Sox10(baz1) protein retains the capacity to contribute to this expression ([13] and this report). To address whether an increase in *neurog1* expression is sufficient to promote DRG neuron specification as in *baz1* mutants, we mis-expressed Neurog1 using the *Tg(hsp70l*:*neurog1)*^*ups1*^ line [60]. When *Tg(hsp70l*:*neurog1)*^*ups1*^ embryos were heat shocked in a WT background a robust increase in DRG sensory neuron cell numbers is detected (Fig 8B and 8C’, quantified in A). We next asked if mis-expression in the absence of Sox10 activity rescues DRG sensory neuron specification. For these experiments we used a morpholino to phenocopy the *sox10* mutant phenotype. This morpholino has previously been shown to phenocopy the pigment cell phenotypes of the *sox10*^*m618*^ allele [61], but its effect on sensory neurons had not been assessed. As expected, injection of the morpholino into WT embryos resulted in a strong decrease in sensory neuron numbers compared with controls (Fig 9D and 9D’, quantified in A). Activation of *Tg*(*hsp70l*:*neurog1*)^*ups1*^ transgene in *sox10* morphant embryos, however, resulted in dramatic rescue of DRG neuron cell numbers (Fig 9D and 9E’, quantified in A); the rescued DRG neurons were frequently arranged as highly expanded DRG, qualitatively phenocopying the *baz1* phenotype. We conclude that in the *baz1* mutants, Sox10(baz1)-dependent activation of *neurog1* expression functions in conjunction with reduced Notch signaling to drive the specification of supernumerary DRG neurons.

## Discussion

In this study, we have comprehensively extended the description of the neural phenotypes of the *baz1* mutants, showing clearly that the glial, as well as enteric and sympathetic neuron phenotypes are similar to other strong loss-of-function *sox10* alleles. Interestingly, the *baz1* sensory neuron phenotype is restricted to the DRGs and does not affect the placode-derived neurons of the cranial ganglia (data not shown). We provide evidence arguing against any changes in the balance of DRG cell proliferation/apoptosis underlying the *baz1* phenotype. Instead, using molecular markers and timelapse studies with double transgenic reporter lines, we note the strikingly precocious and supernumerary nature of that DRG sensory neuron phenotype. These features are typical of defects in lateral inhibition processes, driven by Notch signaling. Previous work in mouse and chick had shown that Delta-Notch signaling controls neuronal versus glial fate choice in the DRGs[42, 43], leading us to test the hypothesis that this mechanism was conserved in zebrafish DRGs. We used two Notch signaling inhibitors (DAPT and compound E) in a 30–72 hpf time-window, combined with transgenic reporter studies to show that Notch signaling is active in the DRGs at these developmental stages. Our expression studies of all known Notch ligands clearly implicate DeltaA and/or DeltaD, and not the other Deltas nor the Jaggeds, as the relevant signals. Although the *jagged* genes’ expression patterns do not overlap with *neurog1*, *sox10* or *foxd3*, making a direct role in DRG development unlikely, the wide expression of *jag1a* in neural cells close to cells expressing these markers, allows the possibility that Jag1a might have some role in DRG development. Indeed, expression of Jagged1 has been documented in mouse DRGs, at the E10.5 developmental stage [62]. However, our studies of the effects of Jag1a knockdown using previously characterised morpholinos [63] resulted in significant increases in *isl1*+ Rohon-Beard neurons but showed no effects on numbers of either *isl1*+ motor neurons nor *sox10*+ neural crest cells in the DRG domain (NT, unpub. data). These data indicate that Notch signaling in the early stages of DRG development depends predominantly on DeltaA and DeltaD. McGraw *et al*. [47] also used DAPT treatment, independently supported by a transgenic approach to inhibit Notch signaling, to assess the requirement for Notch signaling in regulating neuron cell number in larval DRGs. Their treatment windows partially overlapped those used in our study, extending from 2 to 5 dpf, and achieved changes in DRG neuron number quantitatively comparable to those observed in our Notch inhibitor studies described here. McGraw and colleagues demonstrated the expression of *deltaA* and *deltaD* in cells adjacent to differentiating DRG sensory neurons and suggested that Delta-Notch signaling in DRG progenitors controls neuron cell number. Here, we confirm and extend their observations using i) co-expression of *in situ* markers *neurog1* and *neurod1* with *delta* gene expression to show that early neuronal progenitors are expressing *delta* genes, but importantly also ii) by directly demonstrating that activation of Notch signaling is detectable *in vivo* in the early DRGs.

Our study and others’ [47, 64] have left open the possibility of non-autonomous effects of the Notch signaling, but the demonstration here of activated Notch signaling in DRGs, coupled with our and the Raible groups [47] demonstration of expression of Delta-Notch signaling components in these cells, provides strong support for the idea of an autonomous function in the DRGs. Furthermore, recent *in vivo* studies in mouse using a *Wnt1-Cre* transgene to target the neural crest showed an autonomous role for Notch signaling in mouse DRGs[44, 45], so it seems that Delta-Notch signaling in the nascent DRG, and then continuing through larval development as neurons are added in the zebrafish [47], is a conserved mechanism crucial for the balanced production of neurons and glia *in vivo*. Importantly, consistent with this proposal, our direct assessment of Notch signaling levels in the *sox10* mutant DRGs clearly shows that Notch signaling is active in *m618* mutants at levels comparable to WT siblings, but that there is substantial reduction in autonomous signaling activity in *baz1* mutants.

It might seem that a natural interpretation of our *baz1* data is that in this *sox10* mutant allele DRG development occurs in the absence of effective Delta-Notch signaling, perhaps because expression of one or more signaling components is Sox10-dependent and thus compromised in the mutant DRGs i.e. the DRG phenotype is simply a neurogenic phenotype. This conclusion would be consistent with observations from mouse, showing that *Notch-1* expression in nascent DRGs is much reduced in *Sox10*^*Dom*^ homozygotes[20]. But we note that that same study showed that Sox10^Dom^ mutants have a reduced number of DRG sensory neurons compared to WT siblings, and this is consistent with the typical loss-of-function *sox10* phenotype seen in zebrafish [10, 13, 50]. Furthermore, in zebrafish, we show directly here that while Notch signaling is reduced in the developing DRGs of *baz1* mutants, it is apparently *intact* in the more typical *m618* mutant allele. Thus, the molecular explanation for the differential sensory neuron phenotypes is that Sox10 regulates expression of *neurog1* [13], and this is missing in these typical *sox10* mutants, whereas in *baz1* mutants *neurog1* activation by Sox10 is retained and Notch signaling is impaired. Finally, we note that *quantitatively* the effects on DRG sensory neuron numbers of disrupting Delta-Notch signaling using two different drugs (DAPT and Compound E) or a transgenic approach (*c*. 25–30% increase; [47] and this study) are consistently distinct from that of the *baz1* mutant (*c*. 250% increase; [13] and this study). This suggests that the explanation for the *baz1* phenotype must be more than simply loss of Delta-Notch signaling.

In this context, the surprisingly nuanced effects on neural crest development in the unique *baz1* mutant is revealing. These mutants combine a strong loss-of-function phenotype for pigment cells and glia with a gain of function-type DRG sensory neuron phenotype. Our *sox10 in situ* data indicate that a key role for Sox10 in maintenance of its own expression in glial cells is impaired; this is an important observation since Sox10 is a key transcription factor driving glial cell differentiation[20, 31, 65–68]. Thus glial development is largely prevented, consistent with the precocious differentiation of secondary neuromasts shown here. Crucially, however, Sox10-mediated transcriptional activation of *neurog1* is substantially normal in DRG sensory neurons. The subtle substitution (V117M) of hydrophobic residues in the Sox10(baz1) mutant DNA-binding HMG domain would seem compatible with sequence-specific alterations in DNA binding properties, a view supported by our initial *in silico* modelling approaches. These simulations suggested that the *baz1* mutation might result in modifications to the flexibility of the HMG domain structure, but it will require more detailed molecular dynamic and experimental structural analyses to explore the impact of the substitution in detail. For now, we predict that expression of key targets in glial (e.g. *sox10*) and melanocyte (e.g. *mitfa*) development fails as in other loss-of-function alleles, whereas a select subset of targets (e.g. *neurog1*) remain activated at approximately normal levels.

**Fig 10:**
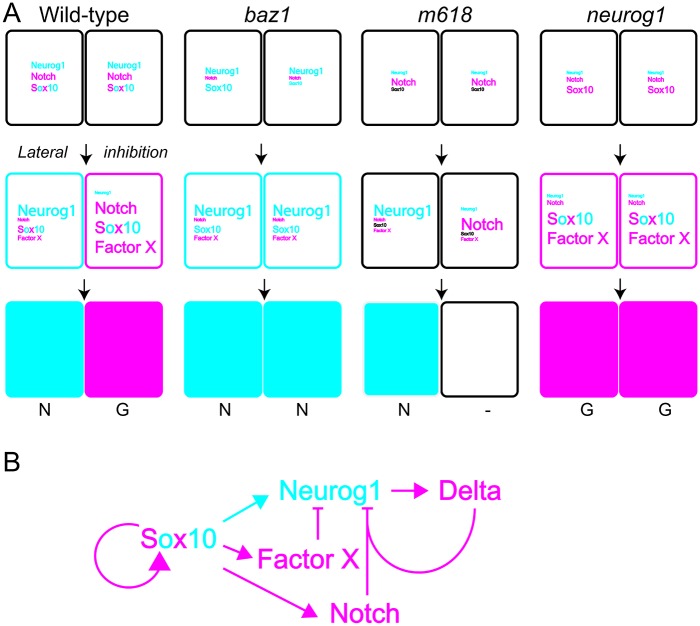
Model of fate specification in DRG development. A) Integrating our data from this study with previous work we suggest how the initial DRG consisting of bipotent DRG progenitors (black rectangles) become specified (second row) and the resultant cell-fates adopted (bottom row). We outline the process in WT (neuron (N, cyan) and glial fates (G, magenta); first column), *baz1* (neuron only; second column), *m618* (failure to undergo fate specification; third column) and *neurog1* mutants (all adopt glia; fourth column). The model summarises at each stage the relative levels of inputs (large font is high activity, small font is weak activity) from each of Sox10, Neurog1, Notch signaling and a Sox10-dependent Factor X, proposed to provide a repressive input on *neurog1* expression. Colour coding of text indicates function of each input (i.e. Neurog1 drives sensory neuron, Notch signaling and Factor X drive glial specification), but Sox10 (which has independent roles in specification of both fates) is shown in both colours where WT, and in blue in *baz1* and in black in *m618* to reflect the neural fate specification activity retained in these mutants. In the case of Sox10 input, note that also font size in neighbouring cells refers to activity with respect to fate being adopted by each cell, since *baz1* mutant reveals that these roles can be separated. The underlying gene regulatory network is outlined in panel B). Asterisk in *m618* cells denotes that sensory neuron specification in *m618* mutants occurs in the absence of Sox10 activity, as a result of Sox9b-mediated transcription of *neurog1*[13]. See text for further details. We note that the data from Fig 8 indicates the possibility that Sox10(baz1) may have unique non-autonomous effects on *neurog1* expression, which, if confirmed, would require modification of the model proposed.

How do these effects combine to generate a neurogenic sensory neuron phenotype in the DRGs? Studies in mouse Sox10 mutants have nicely demonstrated that extracellular signals influencing neural crest cell fate decisions are differentially interpreted depending upon the cellular context and levels of Sox10 protein (and thus, presumably, Sox10 activity)[67]. We propose a model that combines our data with the current understanding of the cellular origin of DRG neurons so as to explain this unique phenotype (Fig 10). An elegant study by the Raible group established that zebrafish DRGs are derived from bipotent sensory neuroglial precursors that express low level *neurog1*[29]. They showed that in normal development a subset of these progenitors maintain and upregulate *neurog1* and thus become sensory neurons, whilst the remaining cells downregulate *neurog1* expression and become glial cells; furthermore, in *neurog1* mutants, all progenitors adopt a glial fate. We note the neurogenic phenotype and recalling its established mechanism, we assume that Sox10 dependent alterations of Delta-Notch signaling are significant. In a WT situation (normal Sox10), Sox10 promotes upregulation of *neurog1* in some precursors as they adopt a neuronal fate. Positive feedback between Neurog1 and Delta expression in the nascent sensory neurons results in enhanced Delta-Notch signaling and, together with Sox10-dependent maintenance of *sox10* expression, this promotes glial fate choice in Notch-responsive cells, achieving a balance of cell-types. In *m618* mutants, Sox10-dependent *neurog1* and *sox10* expression are both impaired, resulting in reduced numbers of sensory neurons and a complete absence of glial differentiation; hence, many DRG progenitors fail to become specified and remain in a progenitor state. Our data shows that Sox10(m618) protein is incapable of activating *neurog1* expression, so the ‘escaper’ neurons are clearly not Sox10-dependent; indeed, in previous work we showed that production of the remaining sensory neurons depends upon Sox9b activity [13]. The number of sensory neurons formed in the *m618* mutant DRGs is restricted to a WT number, likely due to the intact Notch signaling that we show here. In contrast, in *baz1* mutants, Sox10(baz1) retains the ability to activate *neurog1* expression so that sensory neuron specification occurs normally. But in addition, Delta-Notch signaling fails to be activated and Sox10-dependent activation of *neurog1* in neighbouring progenitors in the nascent DRG fails to be repressed. As the neuronal fate is not correctly repressed in putative “glial precursors”, this results in a neurogenic phenotype. As described so far, this would be expected to result in a DRG phenotype equivalent to that generated by inhibition of Notch signaling i.e. a relatively modest increase in DRG neuron numbers. So, how do we explain the elevated numbers of sensory neurons in the *baz1* DRGs? We deduce that Sox10 must have a role in repressing neuronal development in glial progenitors that is independent of Delta-Notch signaling. As noted, in *baz1* mutants, Sox10-dependent positive feedback on *sox10* expression is reduced too, so that glial fates fail to be specified. The *baz1* phenotype demonstrates that one important aspect of this glial fate specification process is a further negative feedback on neuronal development, one that is independent of Delta-Notch signaling but which reinforces Notch signaling-dependent repression of *neurog1* expression (Factor X in Fig 10). Although the molecular details of this mechanism remain to be discovered, the *baz1* phenotype demonstrates that in its absence persistent *neurog1* expression will drive the majority of DRG progenitors to adopt a sensory neuronal fate, consistent with our *neurog1* overexpression data which in both wild-type and *sox10* loss-of-function situations promotes formation of DRGs with strongly elevated sensory neuron numbers. Our study of the effects of over-expressing the different Sox10 proteins (Fig 8) shows unexpected Sox10^-^;Neurog1^+^ cells adjacent to the expected Sox1^+^;Neurog1^+^ cells where Sox10(baz1) was overexpressed. These might be explained by destabilisation of the Sox10(baz1) protein or, more unexpectedly, by an apparent short-range, non-autonomous effect of Sox10(baz1). The former suggestion would be consistent with the general loss-of-function type phenotype displayed by this mutant; in this context, it might be that the ability to maintain activation of *neurog1* would be exceptional.

Our data showing *neurog1* transgene activation by Sox10 proteins (Fig 9) strongly support previous data indicating a direct role for Sox10 in DRG sensory neuron specification via activation of *neurog1* expression[13]. Our transgene data indicate that crucial regulatory elements lie in the region 8.4 kb upstream of the transcriptional start site; consistent with this, we note the presence of multiple Sox10 binding sites in this interval (RM and PB, unpub. data). Further work to characterise the specific binding sites and their regulation will be necessary to fully understand sensory neuron specification. To the best of our knowledge it remains unclear if this mechanism is conserved in mouse [13, 20, 31], although we find conserved Sox10 binding sites upstream of *neurog1* and *neurog2* in mouse.

Together, our data indicates that in the zebrafish DRGs, Sox10 has a crucial role in establishing the ratios of neuronal and glial cell-types, through roles in fate specification of both sensory neurons and glia (via transcriptional activation of *neurog1* expression in nascent neurons and *sox10* maintenance in prospective glial cells, respectively), but also by a mechanism of neuronal fate repression in prospective glial cells that is independent of Notch signaling. A detailed dissection of the molecular mechanisms underlying this intriguing and unexpected role will depend upon extensive characterisation of the structure of the gene regulatory network underlying glial fate specification and differentiation.

## Materials and methods

### Fish husbandry and zebrafish strains

All animals were handled in accordance with the relevant national and international guidelines. Animals in the Kelsh lab are housed in a facility certified by the Home Office, and the work was approved by the University of Bath Animal Welfare and Ethical Review Body and performed under Home Office Project Licenses PPL30/2415 and PPL30/2937. Animals in the Blader lab were housed in a facility certified by the French Ministry of Agriculture: approval ID B-31-555-10. The work in the Blader lab is monitored by a local ethics committee (FRBT C2EA-01). All animals in the Tiso lab were handled under the Italian Ministry of Health authorization n. 407/2015 (UniPD Ethical Committee Reference: OPBA n. 50/2014). Embryos were collected from natural matings and staged (hours post fertilization (hpf) at 28.5°C) according to Kimmel et al. [69]. They were incubated at 28.5°C in 100ml petri dishes containing embryo medium (0.137M NaCl, 5.4mM KCl, 0.25mM Na_2_HPO_4_, 0.44mM KH_2_PO_4_, 1.3mM CaCl_2_, 1.0mM MgSO_4_, 4.2mM NaHCO_3_). Strains used were as described previously: *sox10*^*m618*^ and *sox10*^*baz1*^ [10, 13]; *Tg(neurog1(-8*.*4)*:*nRFP)*^*sb3*^, *Tg(neurog1(-8*.*4)*:*EGFP)*^*sb1Tg*^ and *Tg(neurog1(-3*.*1)*:*EGFP)*^*sb2Tg*^ [49]; *Tg(hsp70l*:*neurog1)*^*ups1*^ [60], *Tg(-4*.*9sox10*:*eGFP)*^*ba2*^ [13], *Tg*(*sox10(7*.*2)*:*mRFP*) [55]; *Tg*(*−2*.*4 kb neurod1*:*GFP*)^*ia50*^ [70]; *Tg*(*12xNRE*:*eGFP*) and *Tg*(*12xNRE*:*mCherry*) [54]. Homozygous mutant embryos were readily identified by pigmentation phenotype.

### Whole-mount in situ hybridisation, antibody staining and TUNEL analysis, imaging and statistical analysis

These were performed as previously described [13], with mutants and siblings in same tube and >20 embryos processed per condition and representative embryos selected for imaging. Experiments were performed at least in duplicate. Fluorescent dual color *In situ* hybridization, based on FastRed/FastBlue double staining, was performed as described in Lauter et al. [71]. *In situ* probes were as follows: *deltaA*, *deltaB*, and *deltaD* [72], *deltaC* [73], *foxd3*[74], *jgd1a*, *jgd1b*, *jgd2* [53], *mbp*[75], *neurog1*[28], *neurod*[28], *notch1a* [76], *notch1b* [77], *notch2* [78], *notch3* [79], *sox10* [10], and *egfp* (Tol2 Kit vector). Antibody staining was largely performed as described in [40]. Prior to primary antibody incubation, embryos were permeabilized with 5μl/ml of proteinase K for 30 mins at 37°C, then rinsed with 5% goat serum at RT for 5 mins and washed 3x1 hour with distilled water at RT. Antibodies used were Hu/Elav1 (1:700 mAb 16A11)[80], Tyrosine Hydroxylase (1:500, Immunostar, Cat. # 22941) and Alexa Fluor 488 goat-mouse IgG (1:750, Molecular Probes, Cat. # A11001). For *post mortem* imaging, embryos were cleared and mounted in glycerol/PBS buffer. For *in vivo* imaging of transgenic lines, embryos were anesthetized in tricaine (0.016%) and mounted in 1% low melting agarose/PBS buffer. Embryos were analysed and imaged using either an LSM510 confocal microscope (Carl Zeiss Imaging) or an Eclipse E800 (Nikon) microscope using appropriate filters and a SPOT digital camera (Diagnostic Instruments) or an AxioImager M2 with Apotome 2 (Zeiss), or using a spectral confocal microscope (Leica SP5), able to selectively acquire near-red fluorescence from FastRed and far-red emission from FastBlue. Image analysis and 3D processing were performed using either ZEN 2009 (Carl Zeiss Imaging) or Volocity 6.0 software (Perkin Elmer). Images were minimally processed using Photoshop (Adobe, Creative Suite 6) to adjust levels, contrast and brightness. Fluorescence quantification was obtained with the Measurements tool of the Volocity 6.0 software, by fluorescent dot counting performed on maximum projection images of selected anatomical regions (ROI dimension: 10 μm x 10 μm x 50 μm; 6 measurements/condition). Counts of DRG cells were performed on 1 side of an embryo only. Statistical analysis was performed using the Prism Statistical Package (GraphPad, San Diego, CA).

### Timelapse studies

For timelapse analysis of DRG development embryos were mounted in 0.6% low melting point agarose in glass-bottomed dishes after dechorionation and anaesthesis in 0.004% tricaine. Imaging was performed using either LSM510META or LSM5 Live confocal microscopes (Carl Zeiss Imaging), with image processing using either ZEN 2009 (Carl Zeiss Imaging) or Imaris 7.1 software (Bitplane A6, Zurich, Switzerland). Embryos were staged by position of PLL primordium [69] at start of experiment. Up to 4 embryos were imaged simultaneously, with wild-type (WT) and *baz1* mutants mounted alongside each other. Images were taken at 30 min intervals for up to c. 25 h.

### Notch inhibitor studies

Notch inhibitor studies were performed on dechorionated embryos (dishes were coated in 2% agarose to prevent adhesion of embryos to dish during treatment. DAPT-treatment was performed according to Geling et al (2002) [57], using 100 μM DAPT in 2% DMSO in embryo medium. Compound E[58] was used in a similar way[81], with embryos treated with 100μM (diluted into 2% DMSO) working solution. In all cases, dishes were wrapped in foil during treatment to exclude light.

### Neurog1 regulation assays

PCR fragments were inserted into a previously described vector to generate Myc-tagged fusion proteins for Sox10(WT), Sox10(m618) and Sox10(baz1) under the control of the *hsp70l* promoter [60]. These plasmids were injected into embryos from the *Tg(neurog1(-8*.*4)*:*EGFP)sb1* line. Embryos were subsequently heat-shocked for 1 hour starting at 7 hpf, and fixed at 9.5 hpf. Finally, *in situ* hybridisations coupled with Myc immunodetections were performed to identify cells expressing the fusion proteins (anti-Myc “9E10”; [82]) and to address whether expression of the reporter transgene (*gfp* mRNA) had been induced in these cells. Three independent injection/induction experiments were performed for each Sox10 variant; at least 40 embryos were injected with each construct. While embryos injected with *hs*:*sox10(m618)* never resulted in ectopic activation of the *neurog1(-8*.*4)*:*EGFP* transgene despite all embryos displaying cells expressing Myc-tagged protein after heatshock, both *hs*:*sox10(WT)* and *hs*:*sox10(baz1)* injection/induction resulted in robust ectopic *gfp* expression. In the case of *hs*:*sox10(WT)* injection/induction, ectopic expression of *gfp* was restricted to Myc-positive cells whereas *gfp*+/Myc-negative cells were often seen adjacent to *gfp+/Myc+* cells when injection/induction was performed with the *hs*:*sox10(baz1)* transgene.

### Molecular modeling

#### 3D prediction

HMG box from human SOX-9 bound to the DNA sequence 5'-CTCTTTGAGAAG-3' has been used as template structure (pdb: 4EUW). Given that wild type and baz1 mutant proteins share 98% sequence identity with the template, backbone and conserved amino-acids have been preserved. Non-conserved amino acids side chains have been reconstructed using the side-chain placement tool SCWRL 4.0 [83].

#### MD simulation

All simulation steps were carried over using the AMBER03 force field [84] within the GROMACS 5.04 (http://www.gromacs.org/) software package [85] for both wild type and baz1 mutant structural models. The simulation was run in a cubic box with a 1.0 nm edge length and the system was solvated with Simple Point Charge water (SPC), a generic equilibrated 3-point solvent model. In order to neutralize the systems, 15 sodium ions were added to replace 15 water molecules. Electrostatic interactions were calculated by the Particle Mesh Ewald (PME) method [86] and All bond lengths were constrained by the LINCS algorithm [87]. Energy minimization was performed and the systems were subjected to a steepest descent energy minimization until reaching a tolerance force of no greater than 1000 kJ mol-1 nm-1. Equilibration of the solvent and ions around the protein-DNA complex was conducted in two phases. The system was slowly heated up from 0 K to 300 K under an NVT ensemble (constant Number of particles, Volume, and Temperature) for 100-ps. After temperature equilibration, pressure stabilization was carried out under an NPT ensemble for 100-ps, wherein the Number of particles, Pressure, and Temperature are all constant and the Parrinello-Rahman barostat was used [88]. The integration time step was 2 fs for both NVT and NPT. Complete MD simulation was run for 100000000 steps (200 ns) at a constant temperature of 300 K and a constant pressure of 1 atm. The trajectories were obtained using “gmx trjconv” GROMACS tool, corrected for periodicity, and analyzed by using “gmx rms” GROMACS utility in order to obtain the Root-Mean-Square Deviation (RMSD). Principal Components Analysis (PCA) has been carried out on the final trajectories by building the covariance matrix of the atomic fluctuations using the “gmx covar”. The set of eigenvectors and eigenvalues was obtained from the diagonalisation of the matrix using “gmx anaeig” tool. The first three principal components representing the largest-amplitude collective motions have been considered.

## Supporting information

S1 Fig Supernumerary sensory neurons in *baz1* mutants express *isl1*. A, B) Expression of *isl1* at 48 hpf in WT (A) and *baz1* (B) is seen in DRGs (*), but is especially prominent in the *baz1* mutants where supernumerary cells are often more ventrally positioned and thus more prominent.
(TIF)

S2 Fig Timelapse movie frames. Extracted frames from S1 video (WT, left) and S2 video (*baz1*, right) of 4 segments of trunk of *Tg(-4*.*9sox10*:*eGFP)*^*ba2*^*; Tg(neurog1(-8*.*4))*:*nRFP)*. Arrows indicate subset of DRG neurons (nRFP^+^). Time since timelapse initiated indicated in Hrs;Mins.
(JPG)

S3 Fig Glial specification is severely defective in *baz1* mutants similar to other *sox10* mutant alleles. A-L) *sox10* expression at 48 hpf. Columns show *sox10*-expressing cells in head (A, E, I), lateral pathway of trunk (B, F, J), medial pathway of trunk (C, G, K) and tail (D, H, L) respectively of 48 hpf WT (A-D), *baz1* (E-H) and *m618* (I-L) mutant embryos. Glia of cranial ganglia and Schwann cell precursors on cranial and PLL nerve (white arrowheads) are prominent in WT, but highly reduced or nearly absent in *baz1* and *m618* mutants respectively. Cells of DRGs (white asterisk) form a prominent segmentally reiterated pattern on the medial pathway of trunk and tail of WT, but are reduced in *sox10* mutants; note that in tail, where cells are developmentally younger, the DRG patterns are more similar. Xanthoblasts (black asterisk) are prominent on lateral pathway of WTs, but reduced and absent in *baz1* and *m618* mutants respectively. M,N,P,Q) *foxd3* expression at 48 hpf. Expression in Schwann cell precursors associated with spinal nerves forms segmentally reiterated pattern readily seen in WT (M), but numbers of cells are much reduced in *baz1* (P). *foxd3*-expressing Schwann cell precursors (white arrowhead) on PLLn are prominent in WT (N), but highly reduced in number in *baz1* at 48 hpf (Q), where they are usually seen only anteriorly. O,R) Supernumerary neuromasts in *baz1* mutants. Whole mount *In situ* hybridisation with *isl1* probe in 48 hpf WT (O) and *baz1* mutant (R). Note supernumerary neuromasts (black arrowheads) in *baz1* mutant. Photos all from single, typical individual of each genotype, after PTU treatment.
(TIF)

S4 Fig Oligodendrocyte differentiation, but not specification, is disrupted in *baz1* mutants. A,B) Oligodendrocyte precursors at 48 hpf in trunk spinal cord express *sox10* and are indistinguishable in number and distribution in WT (A) and *baz1* mutant (B). C,D) *sox10* expression in dispersing oligodendrocyte of hindbrain; note that numbers and dorsoventral distribution strongly resemble wild-type siblings. E,F) *sox10* expression in oligodendrocyte progenitors in the ventralmost spinal cord are unaffected at 72 hpf; region of somites 7–11 is shown. G,H) Oligodendrocyte differentiation is abnormal as shown by strongly decreased *mbp* expression in hindbrain of 72 hpf embryo.
(TIF)

S5 Fig *deltaA* and *deltaD* gene expression overlaps with *neurod1* in the nascent DRGs. A-C) *deltaA* expression (red) is faint, but overlaps with *neurod1* (green) in the nascent DRG (arrows) at 30 hpf. D-F) At 30 hpf, *deltaD* expression (red) overlaps with *neurod1* (green) in the nascent DRG (arrows). G-I) *deltaA* expression (red) clearly overlaps with *deltaD* (green) in the nascent DRG (arrows) at 30 hpf. All main panels are confocal images of fluorescent dual-color *In situ* hybridisations in lateral view, with insets showing *y-z* planes (left) and *x-z* planes (above) for each. nc, notochord; sc, spinal cord.
(TIF)

S6 Fig *m618* and *baz1* Sox10 alleles exhibit different effects on Notch signaling activation. A-D) bright field (A,C) and fluorescent (B,D) views of control (ctrl) and mutant (*m618*, *baz1*) embryos in Notch reporter (NRE:EGFP) transgenic background. *m618* embryos and their controls do not show dramatic differences in Notch reporter activation, while *baz1* mutants exhibit decreased fluorescent signals compared to controls. All panels display 36 hpf embryos in lateral view, anterior to the left. Scale bar: 1 mm.
(JPG)

S7 Fig Transcriptional analysis of Notch reporter expression in *sox10* mutants. A-F) fluorescent WISH analysis in *baz1* and WT sib shows a decrease of *sox10* and Notch reporter transcription in the DRG regions (arrows) of the mutant (D-F), compared to the control (A-C). G-L) fluorescent WISH analysis in *m618* and WT sib shows a decrease of *sox10* and persistency of Notch reporter transcription in the DRG regions (arrows) of the mutant (J-L), compared to the control (G-I). All panels display embryonic trunk regions at 30 hpf, in lateral view with anterior to the left.
(JPG)

S8 Fig Molecular dynamics (MD) simulation of wild type and Sox10(Baz1) HMG box bound to DNA. The results of the principal component analysis of the first three largest-amplitude collective motions are reported. A,B) Energy minimized models of wild type (A) and Sox10(baz1)(B) mutant respectively. Both minimum and maximum extremes of the fluctuations are shown as white ribbons and the average structure is shown as black ribbon. Valine and methionine are highlighted in sticks. C-F) Root mean square deviation (RMSD; *y*-axis) of each structure of the MD trajectory with the corresponding energy minimized structure from 200 ns MD simulation (x-axis time in ns). Panels show the backbone (C,D) and DNA fluctuations (E,F) of wild type (C,E) and Sox10(baz1)(D,F).
(TIF)

S1 video Timelapse of trunk of *Tg(-4*.*9sox10*:*eGFP)*^*ba2*^*; Tg(neurog1(-8*.*4))*:*nRFP)* on WT background from 36 hpf, showing limited numbers of nascent DRG cells (cytoplasmic green) activating *neurog1* reporter transgene (nuclear red). Arrow indicates typical DRG sensory neuron.
(AVI)

S2 video Timelapse of trunk of *Tg(-4*.*9sox10*:*eGFP)*^*ba2*^*; Tg(neurog1(-8*.*4))*:*nRFP)* on *baz1* background from 36 hpf, showing precocious and supernumerary differentiation of nascent DRG cells (cytoplasmic green) into *neurog1*^*+*^ neurons (nuclear red). Arrows indicates subset of DRG sensory neurons.
(AVI)
