# Supplementary figures and images for: Sox10 contributes to the balance of fate choice in dorsal root ganglion progenitors
Source: PLoS One. 2017 Mar 2;12(3):e0172947. doi: 10.1371/journal.pone.0172947 (PMC5333849; doi:10.1371/journal.pone.0172947)

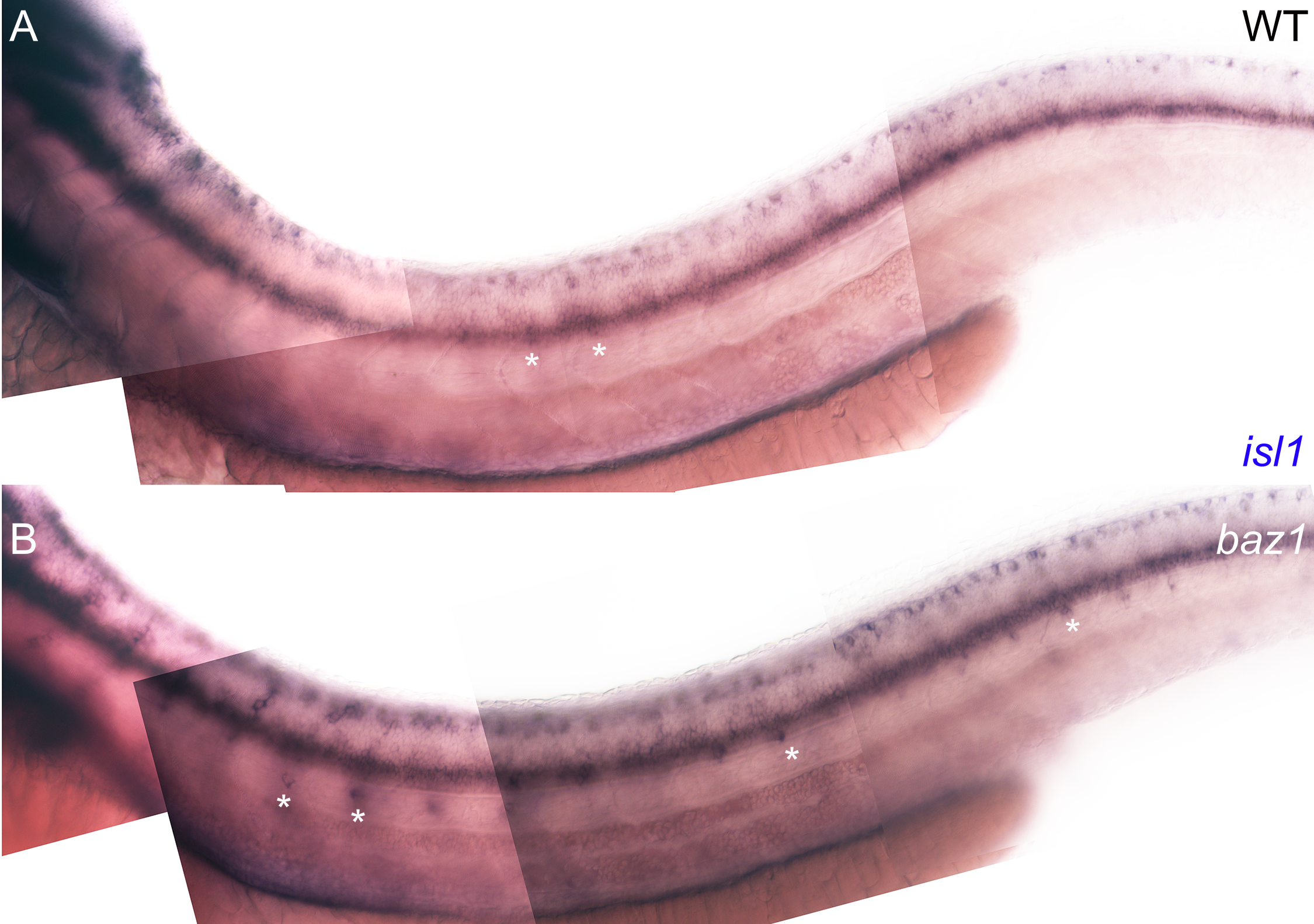

Supplement: S1 Fig — A, B) Expression of isl1 at 48 hpf in WT (A) and baz1 (B) is seen in DRGs (*), but is especially prominent in the baz1 mutants where supernumerary cells are often more ventrally positioned and thus more prominent. (TIF) [file pone.0172947.s001.tif]

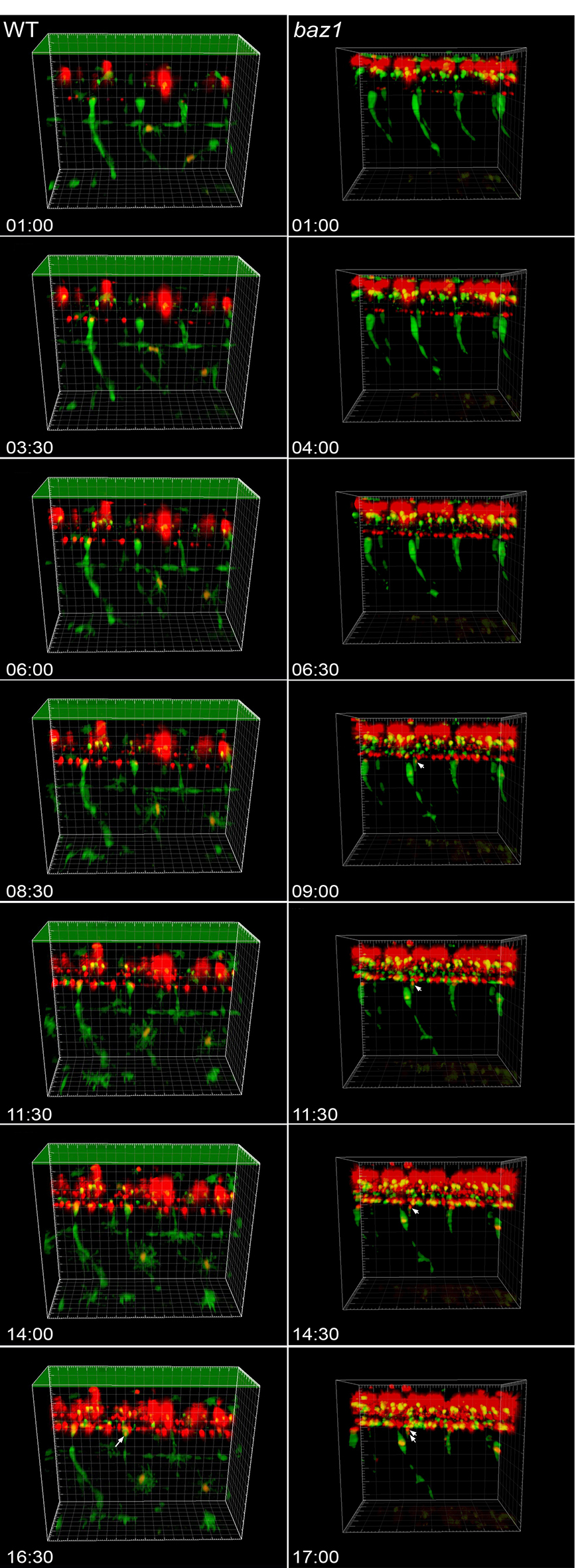

Supplement: S2 Fig — Extracted frames from S1 video (WT, left) and S2 video (baz1, right) of 4 segments of trunk of Tg(-4.9sox10:eGFP)ba2; Tg(neurog1(-8.4)):nRFP). Arrows indicate subset of DRG neurons (nRFP+). Time since timelapse initiated indicated in Hrs;Mins. (JPG) [file pone.0172947.s002.jpg]

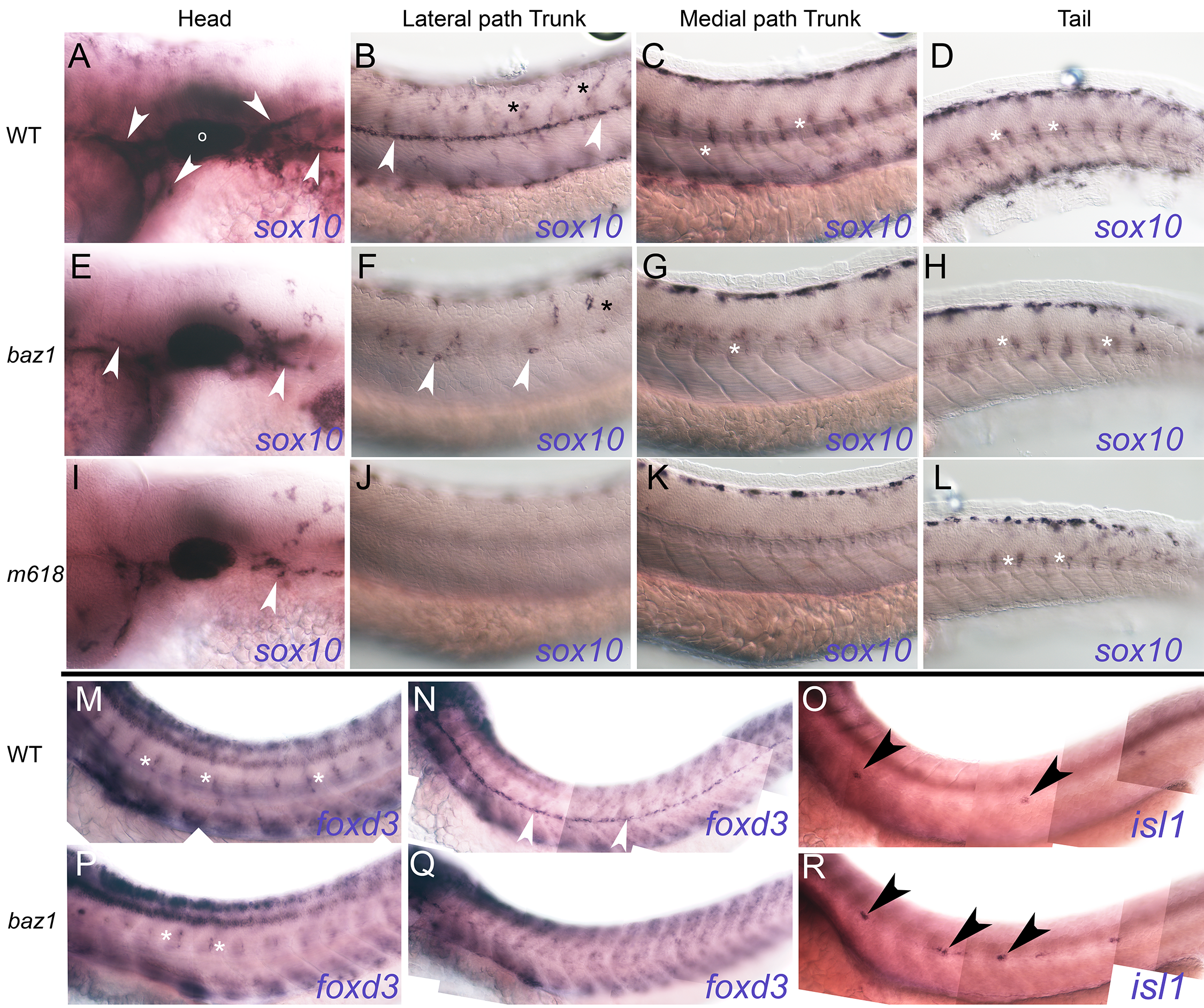

Supplement: S3 Fig — A-L) sox10 expression at 48 hpf. Columns show sox10-expressing cells in head (A, E, I), lateral pathway of trunk (B, F, J), medial pathway of trunk (C, G, K) and tail (D, H, L) respectively of 48 hpf WT (A-D), baz1 (E-H) and m618 (I-L) mutant embryos. Glia of cranial ganglia and Schwann cell precursors on cranial and PLL nerve (white arrowheads) are prominent in WT, but highly reduced or nearly absent in baz1 and m618 mutants respectively. Cells of DRGs (white asterisk) form a prominent segmentally reiterated pattern on the medial pathway of trunk and tail of WT, but are reduced in sox10 mutants; note that in tail, where cells are developmentally younger, the DRG patterns are more similar. Xanthoblasts (black asterisk) are prominent on lateral pathway of WTs, but reduced and absent in baz1 and m618 mutants respectively. M,N,P,Q) foxd3 expression at 48 hpf. Expression in Schwann cell precursors associated with spinal nerves forms segmentally reiterated pattern readily seen in WT (M), but numbers of cells are much reduced in baz1 (P). foxd3-expressing Schwann cell precursors (white arrowhead) on PLLn are prominent in WT (N), but highly reduced in number in baz1 at 48 hpf (Q), where they are usually seen only anteriorly. O,R) Supernumerary neuromasts in baz1 mutants. Whole mount In situ hybridisation with isl1 probe in 48 hpf WT (O) and baz1 mutant (R). Note supernumerary neuromasts (black arrowheads) in baz1 mutant. Photos all from single, typical individual of each genotype, after PTU treatment. (TIF) [file pone.0172947.s003.tif]

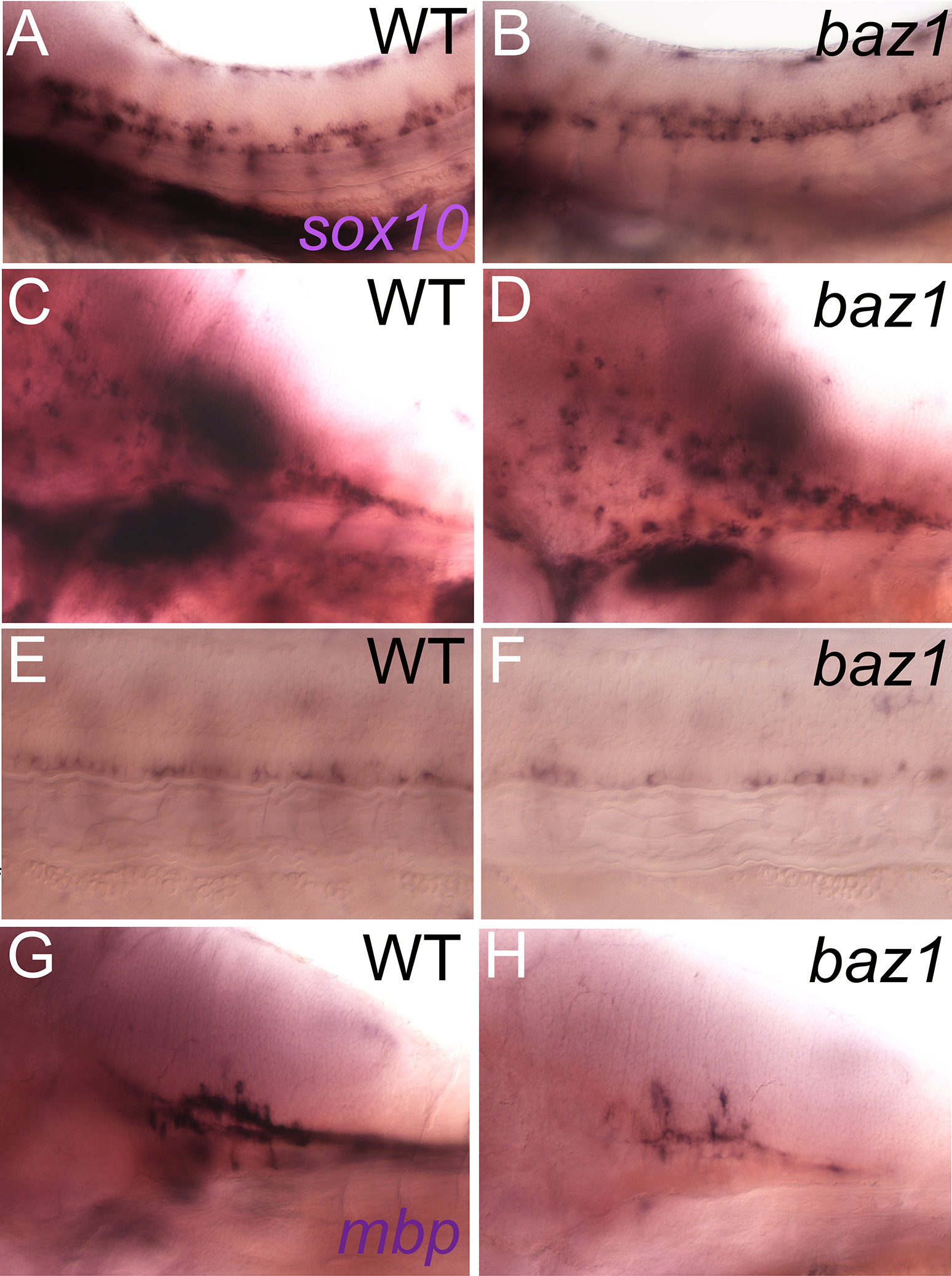

Supplement: S4 Fig — A,B) Oligodendrocyte precursors at 48 hpf in trunk spinal cord express sox10 and are indistinguishable in number and distribution in WT (A) and baz1 mutant (B). C,D) sox10 expression in dispersing oligodendrocyte of hindbrain; note that numbers and dorsoventral distribution strongly resemble wild-type siblings. E,F) sox10 expression in oligodendrocyte progenitors in the ventralmost spinal cord are unaffected at 72 hpf; region of somites 7–11 is shown. G,H) Oligodendrocyte differentiation is abnormal as shown by strongly decreased mbp expression in hindbrain of 72 hpf embryo. (TIF) [file pone.0172947.s004.tif]

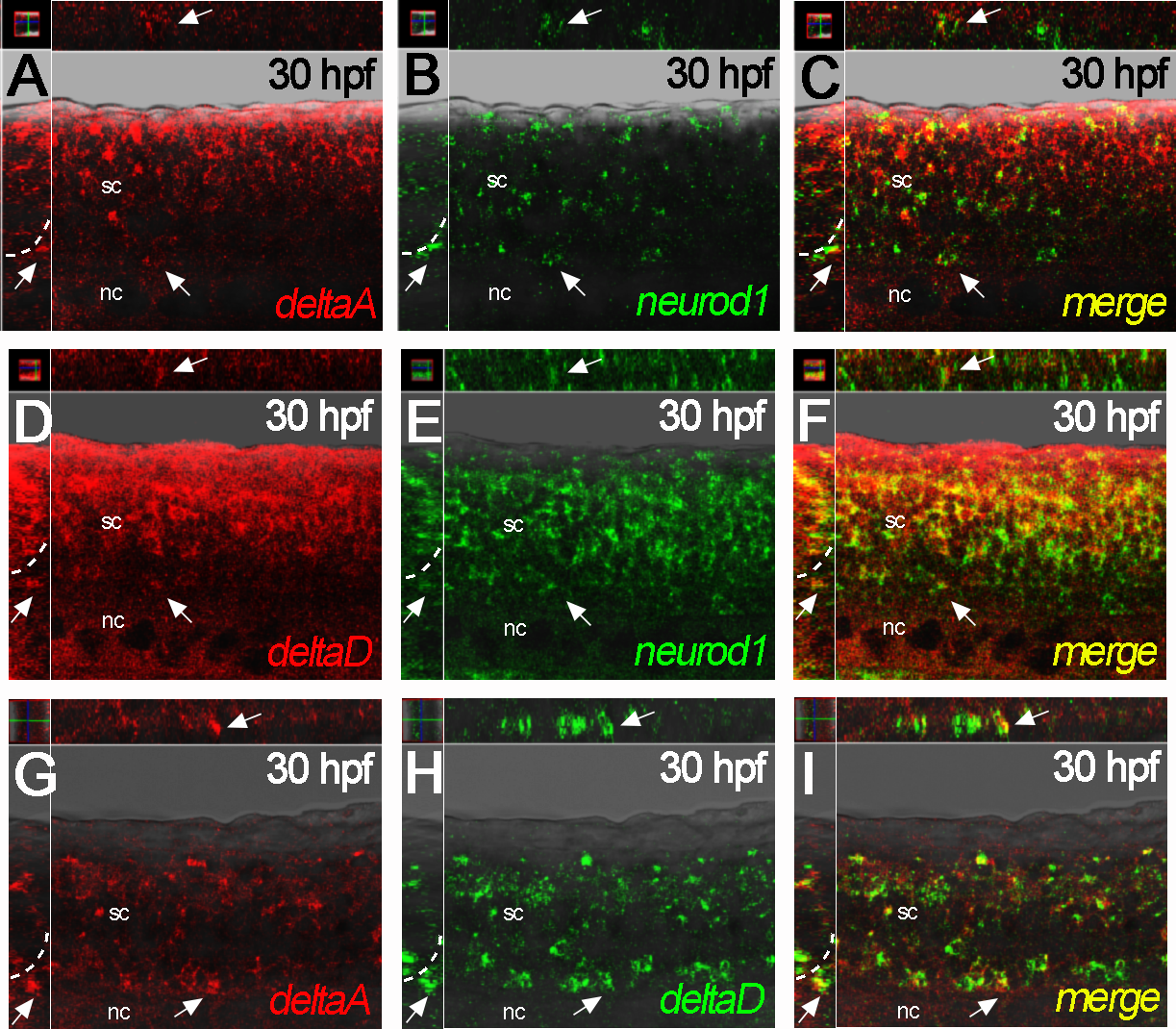

Supplement: S5 Fig — A-C) deltaA expression (red) is faint, but overlaps with neurod1 (green) in the nascent DRG (arrows) at 30 hpf. D-F) At 30 hpf, deltaD expression (red) overlaps with neurod1 (green) in the nascent DRG (arrows). G-I) deltaA expression (red) clearly overlaps with deltaD (green) in the nascent DRG (arrows) at 30 hpf. All main panels are confocal images of fluorescent dual-color In situ hybridisations in lateral view, with insets showing y-z planes (left) and x-z planes (above) for each. nc, notochord; sc, spinal cord. (TIF) [file pone.0172947.s005.tif]

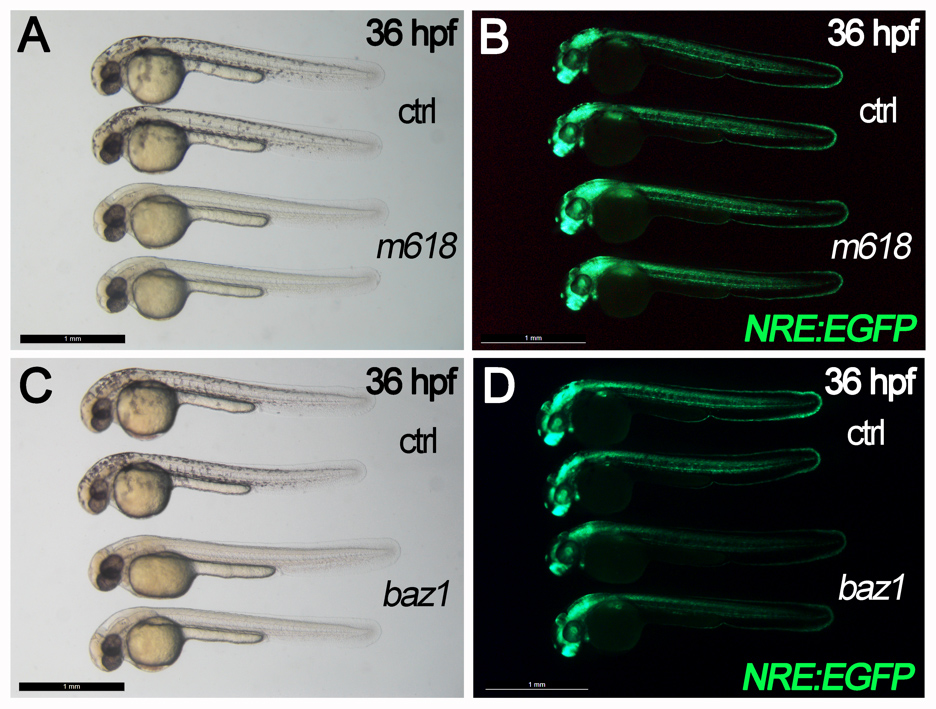

Supplement: S6 Fig — A-D) bright field (A,C) and fluorescent (B,D) views of control (ctrl) and mutant (m618, baz1) embryos in Notch reporter (NRE:EGFP) transgenic background. m618 embryos and their controls do not show dramatic differences in Notch reporter activation, while baz1 mutants exhibit decreased fluorescent signals compared to controls. All panels display 36 hpf embryos in lateral view, anterior to the left. Scale bar: 1 mm. (JPG) [file pone.0172947.s006.jpg]

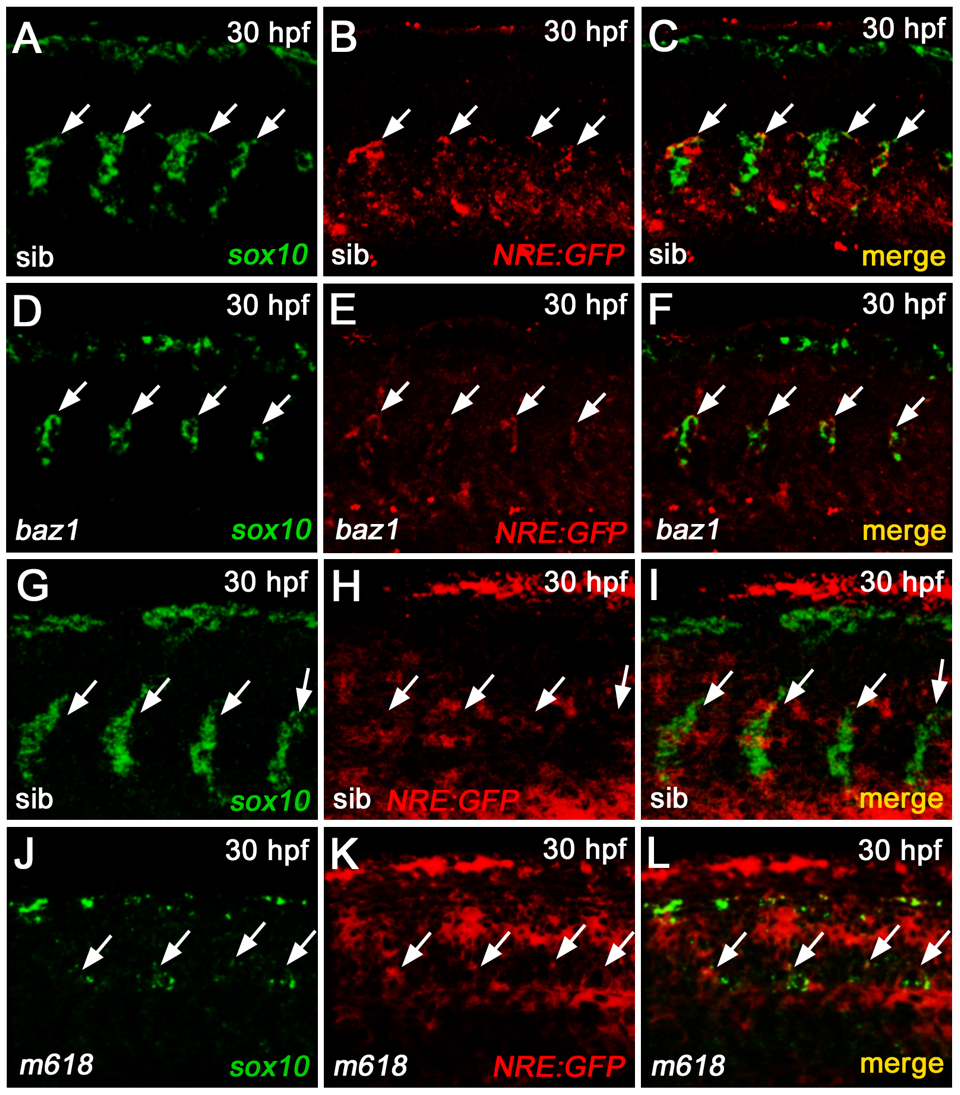

Supplement: S7 Fig — A-F) fluorescent WISH analysis in baz1 and WT sib shows a decrease of sox10 and Notch reporter transcription in the DRG regions (arrows) of the mutant (D-F), compared to the control (A-C). G-L) fluorescent WISH analysis in m618 and WT sib shows a decrease of sox10 and persistency of Notch reporter transcription in the DRG regions (arrows) of the mutant (J-L), compared to the control (G-I). All panels display embryonic trunk regions at 30 hpf, in lateral view with anterior to the left. (JPG) [file pone.0172947.s007.jpg]

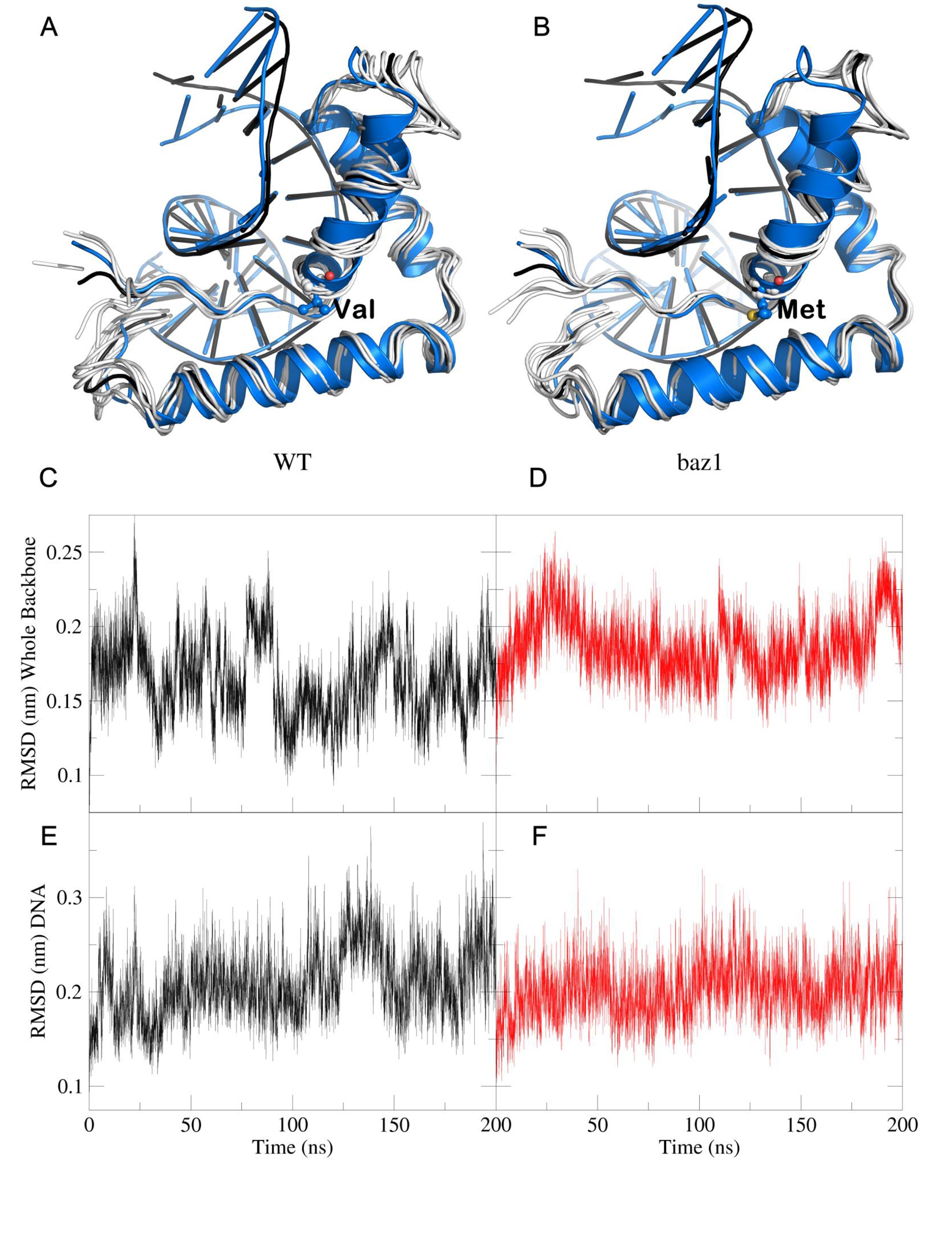

Supplement: S8 Fig — The results of the principal component analysis of the first three largest-amplitude collective motions are reported. A,B) Energy minimized models of wild type (A) and Sox10(baz1)(B) mutant respectively. Both minimum and maximum extremes of the fluctuations are shown as white ribbons and the average structure is shown as black ribbon. Valine and methionine are highlighted in sticks. C-F) Root mean square deviation (RMSD; y-axis) of each structure of the MD trajectory with the corresponding energy minimized structure from 200 ns MD simulation (x-axis time in ns). Panels show the backbone (C,D) and DNA fluctuations (E,F) of wild type (C,E) and Sox10(baz1)(D,F). (TIF) [file pone.0172947.s008.tif]
